# Simple and Versatile Molecular Method of Copy-Number Measurement Using Cloned Competitors

**DOI:** 10.1371/journal.pone.0069414

**Published:** 2013-07-30

**Authors:** Hyun-Kyoung Kim, Hai-Li Hwang, Seong-Yeol Park, Kwang Man Lee, Won Cheol Park, Han-Seong Kim, Tae-Hyun Um, Young Jun Hong, Jin Kyung Lee, Sun-Young Joo, Ju-Young Seoh, Yeong-Wook Song, Soo-Youl Kim, Yong-Nyun Kim, Kyeong-Man Hong

**Affiliations:** 1 Research Institute, National Cancer Center, Ilsandong-gu, Goyang, Korea; 2 Department of General Surgery, Wonkwang University School of Medicine, Iksan, Korea; 3 Department of Pathology, Inje University Ilsan Paik Hospital, Ilsanseo-gu, Goyang, Korea; 4 Department of Laboratory Medicine, Inje University Ilsan Paik Hospital, Ilsanseo-gu, Goyang, Korea; 5 Department of Laboratory Medicine, Korea Cancer Center Hospital, Nowon-gu, Seoul, Korea; 6 Department of Microbiology, Ewha Womans University School of Medicine, Ewha Medical Research Center, Seoul, Korea; 7 Department of Internal Medicine, Seoul National University Hospital, Seoul National University College of Medicine, Seoul, Korea; University of Houston, United States of America

## Abstract

Variations and alterations of copy numbers (CNVs and CNAs) carry disease susceptibility and drug responsiveness implications. Although there are many molecular methods to measure copy numbers, sensitivity, reproducibility, cost, and time issues remain. In the present study, we were able to solve those problems utilizing our modified real competitive PCR method with cloned competitors (mrcPCR). First, the mrcPCR for *ERBB2* copy number was established, and the results were comparable to current standard methods but with a shorter assay time and a lower cost. Second, the mrcPCR assays for 24 drug-target genes were established, and the results in a panel of NCI-60 cells were comparable to those from real-time PCR and microarray. Third, the mrcPCR results for *FCGR3A* and the *FCGR3B* CNVs were comparable to those by the paralog ratio test (PRT), but without PRT's limitations. These results suggest that mrcPCR is comparable to the currently available standard or the most sensitive methods. In addition, mrcPCR would be invaluable for measurement of CNVs in genes with variants of similar structures, because combination of the other methods is not necessary, along with its other advantages such as short assay time, small sample amount requirement, and applicability to all sequences and genes.

## Introduction

Copy-number variations (CNVs) are a quite common polymorphism [1], and have been partially implicated in inter-individual differences in susceptibility to many human diseases. CNVs in *FCGR3B*, *CCL3L1*, and *C4* for systemic lupus erythematosus (SLE) [2], [3], [4], [5], *DEFB4* for Crohn disease [6], alpha-defensin for Psoriasis [7], *CCL3L1* for susceptibility to HIV-1 infection [8], the RHD gene for the Rh-negative blood group [9], and the alpha-globin gene for alpha-thalassemia [10], have been reported. Copy-number alterations (CNAs) are major structural changes in cancer [11], and as such, are predictive markers for cancer patients. *ERBB2* amplification for anti-ERBB2 inhibitor treatment in breast [12] and gastric adenocarcinomas [13], *EGFR* amplification for anti-EGFR inhibitors [14], [15], *MET* amplification for MET tyrosine kinase inhibitors [16], [17], and *FGFR2* amplification for FGFR2 phosphorylation inhibitors [18] have been reported as predictive markers. Integrative analyses of CNAs with gene expression and other molecular changes such as DNA methylation, and microRNA profile, have recently identified new subgroups in breast cancer, which could contribute the management of cancer patients [19], [20].

Array-based methodologies have been used for high-throughput screening of large numbers of CNVs and CNAs. Validation and clinical application of those candidate CNVs and CNAs, however, each require simpler and more accurate methodologies, but have problems in cost, sensitivity, simplicity, and reproducibility. Simple multiplex fluorescent PCR methods have, with acceptable accuracies, been used for validation or clinical application [21], [22], but have been prone to experimental variability. Recently, the Paralog Ratio Test (PRT) has been utilized for accurate measurement of CNVs using repeated DNA elements as internal references, and was successfully adapted to measure CNVs of *FCGR3A* and *FCGR3B* [23]. However, the PRT has limitation in that it is dependent on available dispersed repeat sequences that are similar to the target genes [24]. In addition, for the measurement of *FCGR3A* and *FCGR3B*, an independent Restriction Enzyme Digest Variant Ratios (REDVR) methodology should be combined [23].

Competitive PCR has been employed for precise nucleic acid quantification, and its modified version of competitive PCR employing a modified exogenous sequence has also been applied to the measurement of DNA copies [25]. However, its requirement for meticulously accurate measurements of input genomic DNA amounts greatly hampers its clinical application [25], [26]. Another modified method of the competitive PCR employing multiple modified exogenous reference sequences was applied to measure mRNA quantification, as previously published as real competitive PCR [27]. Real competitive PCR has shown unsurpassed accuracy and reproducibility for quantification of mRNA amounts showing 2-1,000,000 copy differences [27]. However, its application for detection of subtle variation of gene copy numbers has not been easy, and as yet, there have been no reports on its reproducibility or reliability for gene copy determination. In the present study, we modified the real competitive PCR methodology and applied the respective versions to the measurement of gene copy status. We first established our modified real competitive PCR (mrcPCR) method for measurement of *ERBB2* copy status, and the results in primary breast cancer tissues were compared with those for the standard immunohistochemistry (IHC) and fluorescence *in situ* hybridization (FISH) methodologies. Second, we established mrcPCR assays for 24 drug-target genes, used it to determine the copy status in a panel of NCI-60 cells, and compared the results with those for real-time quantitative PCR and a microarray, respectively. Third, an mrcPCR assay was established for simultaneous measurement of CNVs of *FCGR3A* and *FCGR3B*, and the results were compared with those for the combined PRT/REDVR methodology.

## Materials and Methods

### Cell lines and tumor specimens

NCI-60 cell lines were acquired from the National Cancer Institute (MTA No. 2702–09). MDA-MB-453 and JIMT1 cells were purchased from the Korean Cell Line Bank (Seoul, Korea). The institutional review boards of the National Cancer Center and Wonkwang University approved access to and usage of forty-six fresh frozen breast cancer tissues, fifty two control blood genomic DNAs. Institutional review boards waived the need for informed consents in patients whose samples were taken before 2005 according to the Enforcement Decree of Bioethics and Safety Act in Korea.

### DNA isolation

DNAs from the cancer cell lines, fresh frozen breast cancer tissues, and blood cells were isolated using the DNeasy Blood and Tissue Kit (Qiagen, Valencia, CA) and TE buffer (10 mM Tris, 1.0 mM EDTA, pH 8.0) for DNA solubilization. DNA for breast cancer tissues was extracted from 10 to 20 tissue sections (10 μm thick). The cancer cell percentages in the tissues were evaluated using a hematoxylin and eosin (H&E)-stained section. Reference DNA samples from the Research Cell Bank of the Fred Hutchinson Cancer Research Center (IHWG consanguineous panel) were purified again with the DNeasy Blood and Tissue Kit, because the reference DNA samples were contaminated with RNA. The purified DNA stock was maintained in −80°C freezer, and the diluted DNA from the stock using distilled water (Gibco, Carlsbad, CA) was stored at a concentration of 10 ng/μl at −20°C.

To isolate DNA from formalin-fixed paraffin-embedded (FFPE) samples, 2–3 paraffin-embedded tissue sections (20 μm thick) were de-paraffinized, and the tissue was incubated in 1 M sodium thiocyanate overnight at 37°C, followed by Proteinase K treatment at 55°C for about 44 h. Then, genomic DNA was isolated using the DNeasy Blood and Tissue Kit.

### Cloning of competitor DNA sequences used in modified real competitive PCR (mrcPCR)

The competitor sequences were amplified with the primer pairs listed in Tables S2–S4 (Genotech, Daejon, Korea), and the amplified products were cloned into the pGEM-T easy vector (Promega, Madison, WI). The Site-Directed Mutagenesis kit (Stratagene, La Jolla, CA) was used to introduce artificial base changes into the competitor sequences, and the modified bases are listed in Tables S2–S4. The SNP database (http://genome.ucsc.edu/) was consulted to avoid SNP sites in the primer sequences for the PCR or extension reaction. The cloned competitors were digested with restriction enzyme *Sal*I (Fermentas, Glen Burnie, MD), and the mixture of cloned competitors was diluted with distilled water (Gibco) and aliquoted. The competitor plasmids will be available in Addgene (www.addgene.org).

### Establishment of mrcPCR for determination of gene copy number of *ERBB2* or 24 drug-target genes

To determine the *ERBB2* copy status, two *ERBB2* sequences (5-*ERBB2* and 3-*ERBB2*) and two reference sequences (*ALDOC* and *G6PC3*) were utilized as *ERBB2* and reference gene competitors, respectively. To determine the copy numbers for the 24 genes, six multiplex PCR and six extension reactions were performed using five competitors comprising four different drug-target gene sequences and the *IGF1* sequence as a reference. Aliquots of competitor mixtures, primer mixtures, and extension primer mixtures were used to obtain consistent results.

For an mrcPCR assay, the genomic DNA (10 ng) was mixed with competitor mixture containing 10–50 fg of each competitor, and primers (5 pmole), to a final 10 μl volume (primers and modified sequences in Tables S2 and S3). The mixture was then denatured at 98°C for 5 min prior to the PCR. After the extra-denaturation procedure, PCR buffer II (Roche, Mannheim, Germany), dNTP mixture (Roche, final 0.25 mM), MgCl_2_ (Roche, final 2.5 mM), and Taq polymerase (Intron Biotechnology, SungNam, Korea; final 1.5 Unit) in a 10 μl volume was added to the denatured mixture, after which the PCR cycle was performed as follows: denaturation at 94°C for 5 min, followed by 32 cycles of 10 s at 95°C, 30 s at 58°C, and 30 s at 72°C. After the amplification reaction, the amplified products were purified using the AxyPrep PCR Clean-up kit (Axygen, Union City, CA) to remove leftover primers with a final volume of 30 μl. The PCR products were then confirmed in 5% NuSieve agarose (Lonza, Rockland, ME). Subsequently, a single-base extension was performed with the SNaPshot multiplex kit (Applied Biosystems, Foster City, CA) using 2 pmole of extension primers (Tables S2 and S3) and 1 μl of the purified PCR products. The single-base-extension reactions involved 15 cycles of 10 s at 96°C, 5 s at 50°C, and 30 s at 60°C. Alkaline phosphatase (Roche) was then treated for 15 min at 37°C, the product of which was applied to electrophoresis in an automatic sequencer (ABI 3100, Applied Biosystems) with the POP6 polymer (Applied Biosystems).

The mrcPCR method requires only about three hours: 1 hour for extra-denaturation and PCR, 15 min for PCR product purification, 15 min for SNaPshot reaction, 15 min for alkaline phosphatase treatment, 5 min for denaturation, and 30 min for automatic-sequencer analysis. For easier understanding of mrcPCR method, the detailed procedure was described in Methods S1.

### Establishment of mrcPCR for determination of gene copy numbers of *FCGR3A* and *FCGR3B*

For competitive amplification, the *FCGR3* sequence where the nucleotides of *FCGR3A* (C) and *FCGR3B* (T) differ was chosen (C733T, Arginine>Stop), and the base was changed to A for the *FCGR3* competitor. For the *IGF1* competitor, two bases at different sequences of wild-type *IGF1* were changed. In the amplification step, the sequences of *FCGR3* competitor, genomic *FCGR3A*, and genomic *FCGR3B* were amplified competitively, as were the sequences of the *IGF1* competitor and genomic *IGF1*. The competitive PCR was performed under the same conditions, including the extra-denaturation procedure, as for *ERBB2* or the 24 drug-target genes, using the primers listed in Table S4.

The relative amounts of those amplified sequences could be calculated from the relative signals of the extended bases by single-base-extension reaction, which was performed exactly as for *ERBB2* mrcPCR, using the extension primers listed in Table S4.

### Analysis of peak ratios in mrcPCR results

The single-base-extended products were analyzed using a GeneScan software ver.3.7 (Applied Biosystems), and four peak pairs were shown in the mrcPCR results for *ERBB2*. Each peak pair consisted of one signal from the genomic DNA and the other from the competitor. The four peak pairs for *ERBB2* copy status are shown: 1) genomic (A) and competitor (a) peaks of the 3-*ERBB2* sequence; 2) genomic (B) and competitor (b) peaks of the 5-*ERBB2* sequence; 3) genomic (C) and competitor (c) peaks of *G6PC3*; 4) genomic (D) and competitor (d) peaks of *ALDOC*. The signal ratios (SRs: A/a, B/b, C/c, and D/d) for each pair of genomic competitor peak heights were calculated, after which the relative ratios (RRs: Ad/aD, Bd/bD, Cd/cD, Dd/dD  = 1) of the SRs were obtained using *ALDOC* as a reference. Then, the RR values were divided by the mean RR values (aveRR) from 10 control genomic DNA samples in order to obtain the normalized RR (nRR) values representing the copy status of a specific gene. The mean nRR values of 5-*ERBB2* and 3-*ERBB2* relative to *ALDOC* were used for further analysis when there was no apparent change in the nRR of *G6PC3*. When the *ERBB2* copy numbers in tumor tissues were calculated, the tumor fraction determined from the H&E slides was again considered in order to obtain the corrected nRR values, according to the following formula: corrected nRR  =  [nRR – (1– tumor fraction)]/(tumor fraction), where the tumor fraction is the number of tumor cell nuclei divided by the number of total cell nuclei in a H&E slide.

In the copy-number determination for the 24 target genes, five peak pairs were shown, and the peak heights were used to calculate the SRs for each sequence, and were then used to calculate the RRs using *IGF1* SR as a reference, as in the *ERBB2* mrcPCR assay analysis. Then, the RR values were divided by the mean RR values (aveRR) from10 control genomic DNA samples for each gene in order to obtain the normalized RR (nRR) values representing the copy status of a specific gene.

The analysis of the mrcPCR results for *FCGR3A* and *FCGR3B* was performed similarly to that for *ERBB2* or the 24 drug-target genes; the procedure differed somewhat in that two different base ratios were used for *FCGR3* or *IGF1* SRs. The signal peaks of the C (from genomic *IGF1*) and A (from competitor *IGF1*) base-extended EP1 products were used to calculate the SRa for *IGF1*, and the signal peaks of the T (from genomic *IGF1*) and A (from competitor *IGF1*) base-extended EP2 products were used to calculate the SRb for *IGF1*. The signal peaks of the C (from genomic *FCGR3A*) and A (from *FCGR3* competitor) base-extended EP3 products were used to calculate the SRa for *FCGR3A*, and the signal peaks of the T (from genomic *FCGR3B*) and A (from the *FCGR3* competitor) base-extended EP3 products were used to calculate the SRb for *FCGR3B*. The SRa for *IGF1* was then used to calculate the RRa value for *FCGR3A*, and the SRb for *IGF1* was used to calculate the RRb value for *FCGR3B*. Since control samples are not available, the most common alleles of *FCGR3A* or *FCGR3B* were assumed to have two copies, as reported previously [23]. Accordingly, the median RR values for *FCGR3A* and *FCGR3B*, excepting the samples for which they were high or low outliers, were employed for the normalization, and the resultantly normalized RR (nRR) values were used for further analysis.

To compare the results by mrcPCR with those by PRT/REDVR, the sum of the nRR values of *FCGR3A* and *FCGR3B* (nRRa+b), and the ratio of the nRR values of *FCGR3B* to *FCGR3A* (nRRb/a) were used.

### Absolute quantification of *FCGR3A* and *FCGR3B* copies by mrcPCR using cloned plasmids

When the reference-gene-copy number is known, the absolute copy number of the target gene could be deduced from the standard curve of the RR values from the mixtures of the cloned target and reference plasmids. The *FCGR3A*, *FCGR3B*, and *IGF1* sequences were amplified with PCR primers (Table S4), and the plasmids were digested with *Sal*I, as the competitor treatment. After determining the concentration of the purified plasmids using the Quant-iT PicoGreen dsDNA Reagents (Invitrogen, Eugene, OR), each plasmid was diluted into 50 ng/μl. The diluted *FCGR3A* or *FCGR3B* plasmid was admixed in various ratios with the *IGF1* plasmid to a final volume of 10 μl. Then, each mixture was diluted 10^6^-fold to a final *IGF1* plasmid concentration of 10 fg/μl. The diluted mixture was employed in the mrcPCR in place of genomic DNA sample under the same conditions. To calculate the RR values, the same formulas were used, because the same base-extended EP3 products as for the genomic DNA samples appeared in the electrophoregram. Based on the RR values for each plasmid mixture, the standard curve was plotted as shown in Figs. S2A and S2B for *FCGR3A* and *FCGR3B*, respectively.

### Microarray Analysis

A HumanCytoSNP-12 microarray (Illumina, San Diego, USA), as performed by SNP Genetics (Seoul, Korea), was used to determine the copy numbers in the COLO205 and OVAR-4 cells. In the analysis, 200 ng of DNA was used as the input for a single array. DNA amplification, tagging and hybridization were performed according to the manufacturer's protocol, and the array slides were scanned on an iScan Reader (Illumina).

For the comparison with the mrcPCR results, the average copy-number values for six SNP sites located within or near the specific genes in the microarray results were used, because the signal variation from the microarray data was relatively high for each individual SNP site.

### Determination of *ERBB2* status by immunohistochemistry and/or fluorescence *in situ* hybridization

Immunohistochemical (IHC) results from hospital record were used. When no hospital record of IHC result was available, IHC staining was performed after preparation of FFPE tissues from fresh frozen cancer tissues. Borderline cases (2+ in IHC) were analyzed by FISH (fluorescence *in situ* hybridization). Scoring for IHC and FISH was performed according to ASCO guidelines [28].

### Quantitative real-time PCR for copy-number determination

Quantitative real-time PCR (LightCycler 480, Roche, Indianapolis, IN) using the SYBRGreen Reagent Kit (Roche) with the primers listed in Table S5 were performed to determine the *PTK2*, *MYC*, and *FGFR1* copy numbers using *IGF1* as a reference gene and 10 ng as the input sample amount, as follows: denaturation at 94°C for 5 min, followed by 40 cycles at 94°C for 10 s, 55°C for 10 s, and 72°C for 30 s. The copy number was calculated according to the fold changes relative to the normal control (2_DDCt), as previously described [29].

### Measurement of *FCGR3A* and *FCGR3B* copy numbers by PRT and REDVR

The PRT and REDVR assays were performed as previously reported [23], excepting minor modifications. In order to obtain more stable amplification products for both assays, modified longer primers were used (Table S6). The REDVR PCR products were purified using the AxyPrep PCR Clean-up kit (Axygen, Union City, CA), digested with *Taq*I, and then analyzed on the ABI sequencer. The PRT PCR products were diluted 100-fold, and analyzed on an ABI sequencer as previously reported.

In order to incorporate an extra-denaturation procedure into the PRT and REDVR assays, sample DNA and primers in a 10 μl volume were denatured at 98°C for 5 min. After the denaturation, the other components were mixed to a final volume of 20 μl, and amplification was performed as previously reported [23].

The *FCGR3A* and *FCGR3B* copies were calculated from the PRT ratio and REDVR b/a, the PRT ratio being the ratio of the peak area of *FCGR3* to that of the reference sequence on chromosome 18, and the REDVR b/a, the ratio of the peak areas of 185-bp *FCGR3B* to 136-bp *FCGR3A*. The *FCGR3A* copy was calculated by the formula (PRT ratio)/(1+ REDVR b/a), and the *FCGR3B* copy by (REDVR b/a)*(PRT ratio)/(1+ REDVR b/a). Whereas the *FCGR3A* copies calculated from the combination of PRT/REDVR methods were directly used, the *FCGR3B* copies were normalized using median *FCGR3B* copy value in the tested samples excepting high or low outliers.

### Statistical Analysis

The statistical significance of the difference between the Log R ratio by microarray and the nRR of the 24 drug-target genes by mrcPCR was tested by Pearson correlation after logarithmic transformation of the nRR values to normalize the distributions. The significance of the difference between the quantitative real-time PCR and mrcPCR results was tested by Pearson correlation after logarithmic transformation. nRRa+b and the PRT ratio were compared by Pearson correlation test after logarithmic transformation of the values from both methods. REDVR b/a and nRRb/a were compared by Pearson correlation test after logarithmic transformation of the values from both methods. Finally, the correlation for two types of assays or for two measurements of a single assay was tested by Pearson correlation after logarithmic transformation of the values.

## Results and Discussion

**Figure 1:**
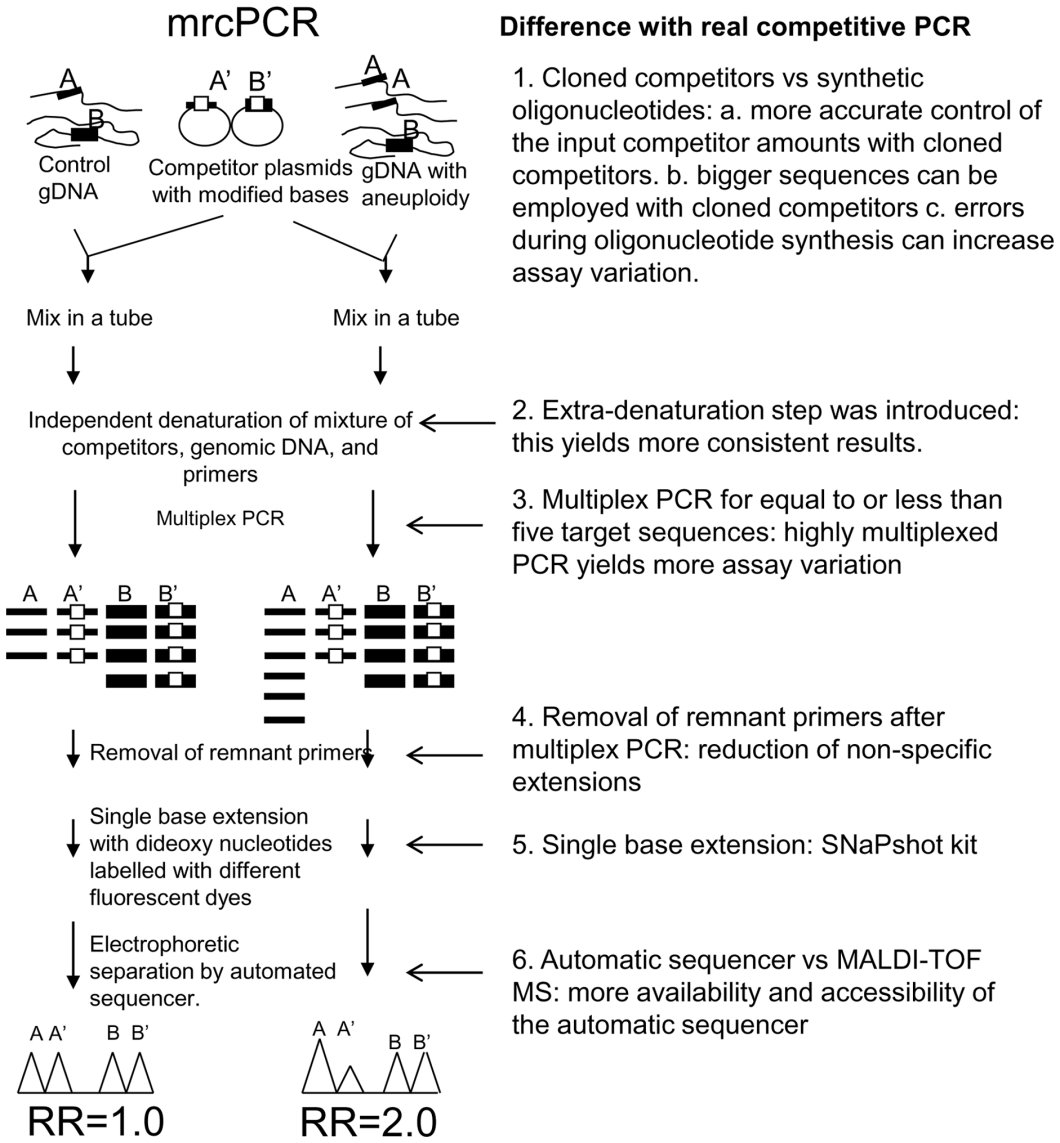
Schematic diagram of mrcPCR showing its differences with real competitive PCR. On the left side, the scheme of mrcPCR is shown. On the right side, the differences are summarized. In the result analysis, the relative ratio (RR) of the signal ratios (SRs) of A/A' and B/B' [RR  =  (A/A')/(B/B')] was used, representing the relative copy status of target gene A compared with reference gene B. A or B is the genomic peak height from the A or B gene. A' or B' is the competitor peak height from the A' or B' competitor. In the example, the test sample has two times more of A gene than reference sample, and so the final RR value for the A gene is 2.0, when B and B' are the same. In most cases, B and B' are different, so control samples are needed to normalize the ratio of B to B' and get normalized RR (nRR).

In the present study, we modified the real competitive method [27], and tested whether the modified real competitive PCR (mrcPCR) could be applied to gene copy determination. The principles of real competitive PCR and mrcPCR are similar in that they both entail the use of competitor sequences with an artificially introduced base. However, the mrcPCR embodies modifications for measurement of subtle copy-number difference: Cloned competitors instead of synthetic oligonucleotides, and an automatic sequencer instead of MALDI-TOF MS spectrometry, are employed. Thus in mrcPCR, longer competitor sequences can be employed for more reproducible competitive amplification, and the use of a more widely available automatic sequencer promises to enhance the accessibility of this method. Other modifications, including the inclusion of an extra-denaturation procedure and the removal of remnant PCR primers prior to single-base-extension reaction, were introduced for accurate gene copy assessment. The mrcPCR and its differences with real competitive PCR are schematically presented in Figure 1.

### Measurement of *ERBB2* amplification by mrcPCR in breast cancer tissues

**Figure 2:**
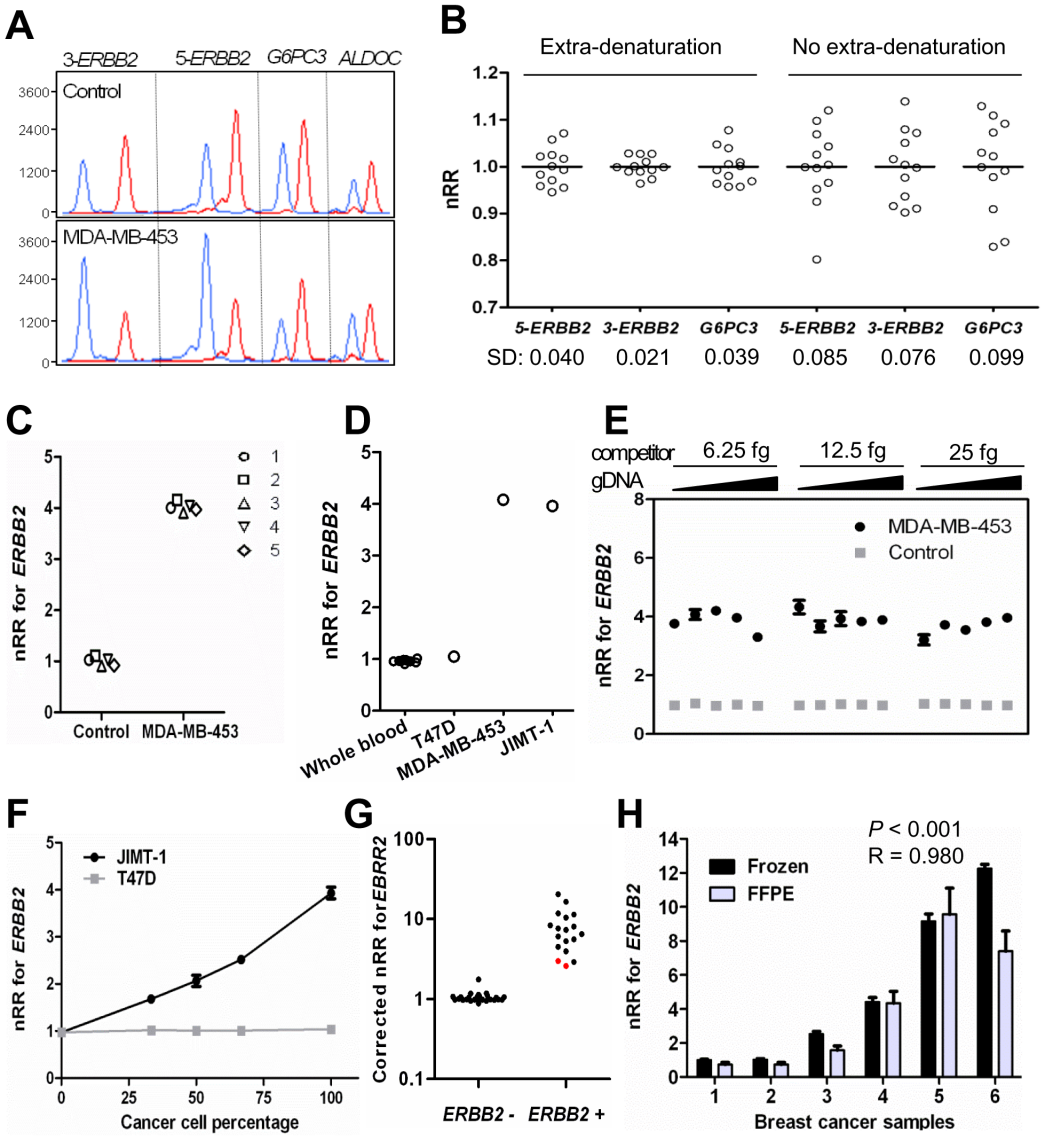
Measurement of *ERBB2* gene copy number by mrcPCR. (A) Representative results for measurement of *ERBB2* copy number by mrcPCR using two *ERBB2* sequences (5-*ERBB2* and 3-*ERBB2*) and two reference sequences (*ALDOC* and *G6PC3*). Eight peaks originated from either genomic DNA (blue) or the competitor (red). (B) mrcPCR assay variation depending on extra-denaturation procedure. Standard deviation (SD) values for the nRRs are shown. (C) Reproducibility of mrcPCR by five independent assays. (D) *ERBB2* copy number by mrcPCR in control and cancer cell samples. The y-axis shows the mean nRR values of 5-*ERBB2* and 3-*ERBB2*. (E) mrcPCR results using various amounts of input genomic DNA (blue, 2.5, 5, 10, 20, or 40 ng) and competitors (red, 6.25, 12.5, or 25 fg based on *ALDOC* competitor). The y-axis shows the mean nRR values of 5-*ERBB2* and 3-*ERBB2*. (F) Detection of *ERBB2* amplification in serially-diluted DNA of JIMT-1 or T47D cells. The y-axis shows the mean nRR values of 5-*ERBB2* and 3-*ERBB2*. The assay was performed in duplicate. (G) Comparison of mrcPCR results with those from standard methods of immunohistochemistry (IHC) and fluorescence *in situ* hybridization (FISH). Y axis: corrected nRR for *ERBB2* copy based on cancer cell percentage as described in Materials and Methods. The red spots are the samples that contained only about 30% tumor cells. (H) Comparison of mrcPCR results between fresh-frozen (black) and formalin-fixed paraffin-embedded (FFPE, gray) cancer tissues (*P*<0.001, R = 0.980, by Pearson correlation test after logarithmic transformation). The assay was performed in triplicate.

Because of the availability of the standard IHC and/or FISH methods for *ERBB2* copy status determination [28], the mrcPCR assay for this gene was established. In the assay, two sequences of *ERBB2* (marked 5-*ERBB2* and 3- *ERBB2*) were employed along with two reference genes, namely *G6PC3* and *ALDOC*, in 17q (Fig. 2A). Cloned competitors for the two *ERBB2* sequences, and two reference genes were digested with restriction enzyme, owing to the fact that employment of the undigested competitor plasmids for mrcPCR yielded inter-assay variations, probably due to the plasmid structural variations between the relaxed and closed circular forms. In order to reduce the effects of plasmid structural variation, an extra-denaturation procedure prior to the competitive PCR was used as well.

In the mrcPCR assay, the signal ratios (SRs) of the competitor and corresponding wild-type sequences varied with the varying input ratios of the sample DNA and competitor amounts: when the input amount of the sample was small, the relative amplified amount of the wild-type sequence was smaller, and the resulting SR for *ERBB2*, correspondingly, was lower. In order to obtain more stable SRs, the sample DNA concentration was pre-adjusted to 10 ng/μl prior to initiating the mrcPCR assay. In normal-blood DNA samples, the standard deviation (SD) errors in the nRR values (normalized with mean RR values) were 0.076–0.099, as shown in Fig. 2B. Although the mrcPCR assay has its advantage in yielding multiple gene copy information, the SD error value of the mrcPCR was barely better than that from real-time quantitative PCR, which is about 0.21 [30]. However, when the extra-denaturation procedure was introduced for DNA samples along with the competitors, the SD error was markedly reduced, to as small as 0.021–0.040, as shown in Fig. 2B (SD for nRR of 5-*ERBB2*/*ALDOC*  = 0.040; SD for nRR of 3-*ERBB2*/*ALDOC*  = 0.021; SD for nRR of *G6PC3*/*ALDOC*  = 0.039). These results suggest that the mrcPCR can determine *ERBB2* gene copy status more reliably with the extra-denaturation procedure. We performed the extra-denaturation procedure on a mixture of competitors, genomic DNA, and primers just before the PCR for all of the mrcPCR assays. With the extra-denaturation procedure, five independent experiments using DNAs from normal whole blood or MDA-MB-453 showed consistent results (Fig. 2C).

*ERBB2* amplification, as expected, was detected in MDA-MD-453 and JIMT-1 cells, but not in T-47D (Fig. 2D). However, the relative copy numbers of the reference genes (*G6PC3* or *ALDOC*) were not 1.0 in either cell line, probably due to co-amplification in one of the reference gene loci along with the amplification of *ERBB2*. For example, the MDA-MB-453 cells had a higher copy number for *ALDOC* than for *G6PC3*, and the JIMT-1 cells had a higher copy number for *G6PC3* than for *ALDOC*, suggesting that the use of one reference gene close to *ERBB2* could under-estimate the *ERBB2* copy number. So, in the present study, the reference gene with the lower nRR value, either *ALDOC* or *G6PC3*, was used to evaluate the *ERBB2* status, when there is apparent difference in nRR values of reference genes.

In previously reported competitive methods, exact amounts of input competitors and sample DNAs were essential [25], [26], and so clinical application was difficult. However, in our present mrcPCR assay, the use of various amounts of genomic DNA (2.5–40 ng) or competitor mixture (6.25–25.0 fg) yielded consistent results (Fig. 2E), indicating that meticulous measurement of the DNA concentration or the addition of exactly the same ratio of sample DNA to competitors is not necessary. Furthermore, the input ratios between competitor and genomic DNA could be monitored by SRs, thus ensuring detection of possible experimental errors.

Cancer tissues usually contain normal cell components, which limits the sensitivity of molecular methods; mrcPCR shares this limitation. However, in an assay using different mixture percentages between the reference blood gDNA and JIMT-1 gDNA, the mrcPCR could reliably detect *ERBB2* copy gain in samples containing more than 33% *ERBB2*-amplified gDNA of JIMT-1 (Fig. 2F). Moreover, the relationship between the nRR values by mrcPCR and the relative amounts of JIMT-1 gDNA was linear (Fig. 2F). These results suggest that the mrcPCR can be effectively used for cancer tissues containing admixed normal cell components, and that cancer-cell-percentage-based linear correction can be possible.

In a comparison of the mrcPCR results with those by the standard IHC and/or FISH methods, the results were consistent across 46 breast cancer tissues, excepting two cases with low tumor percentages of about 30% (marked in red, Fig. 2G). However, all of the results were consistent when the nRR values were corrected relative to the tumor percentage (Fig. 2G).

As most available clinical samples are formalin-fixed paraffin-embedded (FFPE), not fresh-frozen cancer tissues, the applicability of mrcPCR to FFPE tissues was tested for six sample pairs of both frozen and FFPE tissues. The correlation between the results was significant (*P*<0.001 and R = 0.980, by Pearson correlation test after logarithmic transformation, Fig. 2H), suggesting the clinical applicability of mrcPCR to FFPE tissues. However, these FFPE samples were freshly prepared from fresh-frozen tissues, and were less than two weeks old at the time of DNA preparation; and in fact, the results for subsequently-tested several-years-old FFPE tissues proved less reliable. Nonetheless, mrcPCR can be considered to be applicable in routine practice, given that freshly prepared FFPE tissues normally are available.

Thus, we showed that the mrcPCR assay can determine *ERBB2* copy status with sufficiently high sensitivity and reproducibility in cancer cells and tissues.

### Measurement of 24 drug-target gene copies in panel of NCI-60 cells by mrcPCR

**Figure 3:**
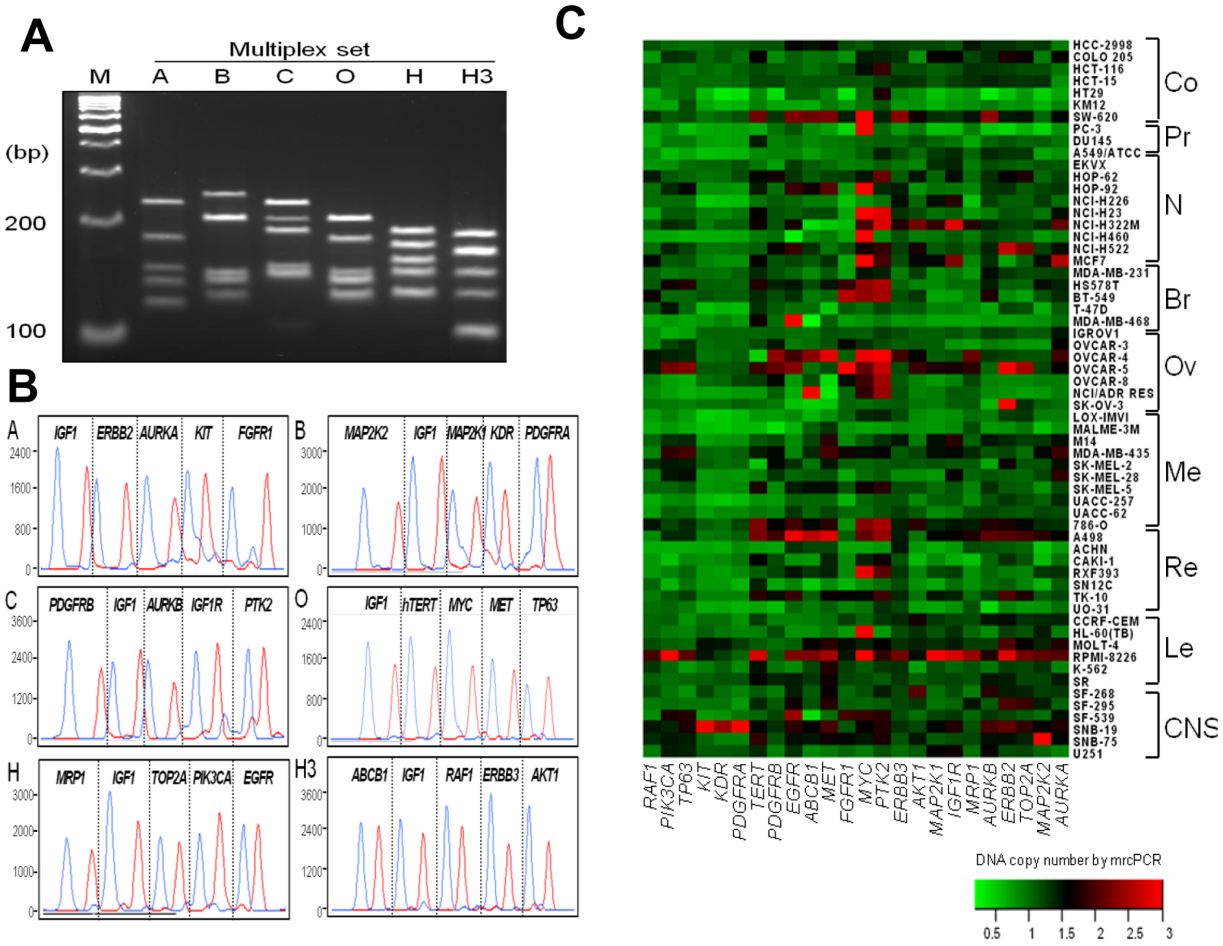
Measurements of copy numbers for 24 drug-target genes by mrcPCR in NCI-60 cells. (A) PCR amplification products from each of six multiplex PCR (A, B, C, O, H, and H3) separated by 5% NuSieve® agarose gel electrophoresis. (B) Representative peaks from analysis of products of six single-base-extension reactions (A, B, C, O, H, and H3) in a control whole-blood DNA sample. Five peak pairs are shown for four target genes and one reference gene in each reaction. Each pair of peaks is from either genomic DNA (blue) or the competitor (red). (C) Heatmap of copy numbers for 24 genes in NCI-60 cells. Each copy number was measured three times and the mean values were used. Co, colon; Pr, prostate; N, NSCLC; Br, breast; Ov, ovarian; Me, melanoma; Re, renal; Le, leukemia.

In clinical application of CNAs as prognostic and/or predictive markers for cancer patients, the copy status of dozens of genes may be used simultaneously, as there are many targeted inhibitors available even for a single cancer type. To accommodate such needs, mrcPCR method was developed for 24 drug-target genes (Table S1) selected based on the availability of specific targeted inhibitors [31]. The method consisted of six multiplex PCRs and six single-base-extension reactions. The representative assay results for a control blood DNA sample are shown in Figures 3A and 3B, and the copy numbers for the NCI-60 cell lines as determined by mrcPCR are mapped in Figure 3C.

**Figure 4:**
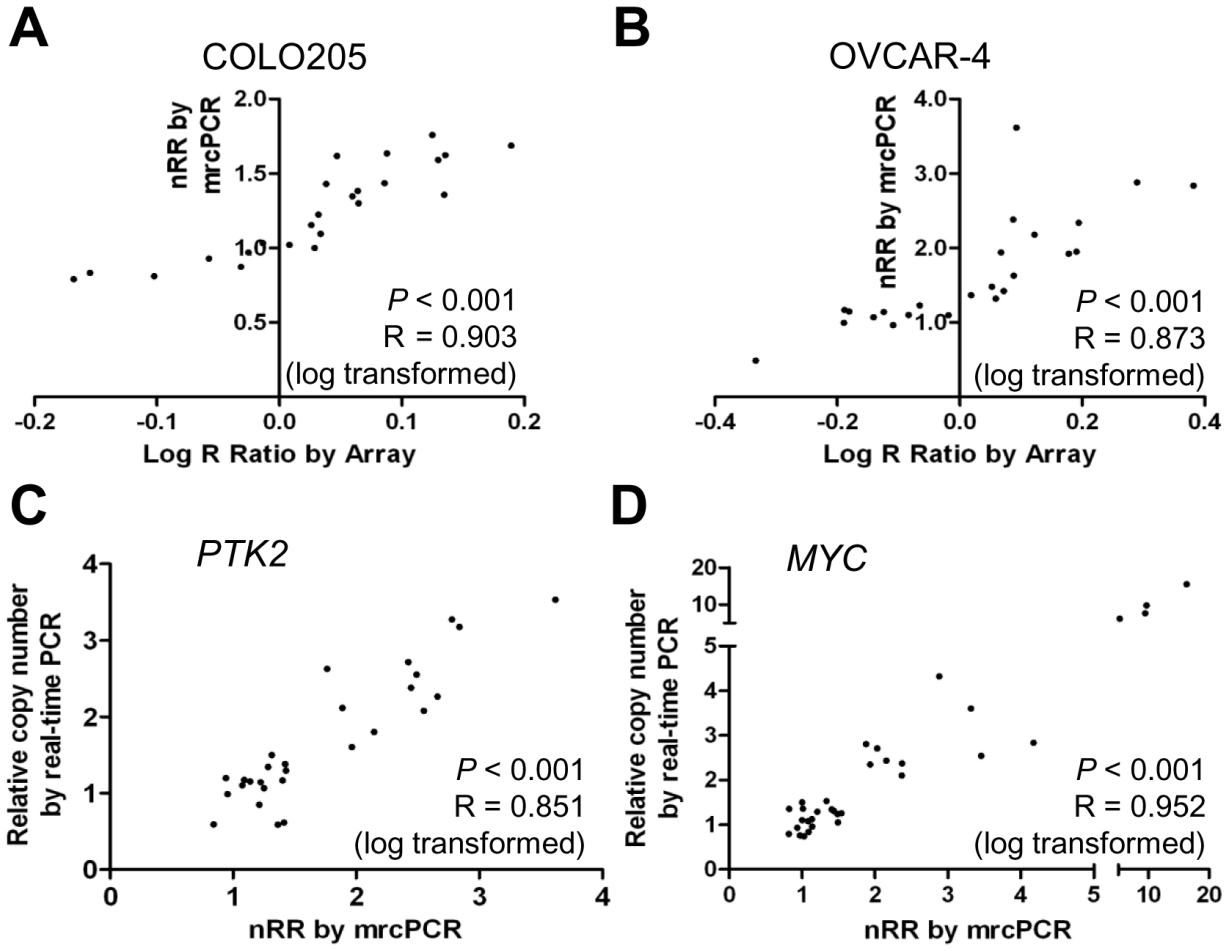
Validation of mrcPCR assay with microarray or real-time PCR. Comparison of results between mrcPCR and microarray in COLO205 (A), and OVCAR-4 (B) cells. Comparison of results between mrcPCR and real-time PCR for *PTK2* (C) and *MYC* (D). The Pearson correlation coefficients (R) and *P* values after logarithmic transformation are shown.

To confirm the reliability of the copy status determination for the 24 genes, these mrcPCR results were compared with those by microarray or real-time quantitative PCR. In an analysis utilizing COLO205 and OVAR-4 cells, the correlation was significant (*P*<0.001 for both cells; R = 0.903 for COLO205 and R = 0.873 for OVCAR-4, by Pearson correlation test after natural logarithmic transformation of nRR values; Figs. 4A and 4B). In comparison with a real-time quantitative PCR on two genes, *PTK2* and *MYC*, the copy numbers of which were changed most frequently in the NCI-60 cells, the results were highly consistent (*P*<0.001 for both genes; R = 0.851 for *PTK2* and R = 0.952 for *MYC*, by Pearson correlation test after logarithmic transformation of nRR values; Figs. 4C and 4D). To demonstrate reliable detection of copy loss, the comparison of *FGFR1* results between mrcPCR and real-time PCR was tested as well (Fig. S1). These data indicate the reliability of the mrcPCR results for the 24 genes.

Although the mrcPCR could determine the copy status effectively in most of the NCI-60 cells, the copy numbers in the RPMI-8226 leukemia cells were relatively high for most of the 24 genes tested (median nRR of 24 genes  = ∼2.2). The reason might be the copy loss of reference gene *IGF1*, but we did not investigate further. This possibility indicates that application of the mrcPCR method requires the simultaneous use of several reference genes. However, employing several reference genes for each assay could reduce the versatility of mrcPCR, because less than six genes seems to be the optimal number in a single mrcPCR assay for accurate determination of gene copy numbers. Alternatively, therefore, we herein propose the use of a separate panel consisting of several reliable reference genes for determination of baseline copy status: genes showing in certain types of cancer relatively stable copy numbers as deduced from microarray experiments or multi-copy repetitive sequences (as utilized in human multicopy reference PCR assays from Qiagen) can be employed as mrcPCR reference genes.

### Measurement of *FCGR3A* and *FCGR3B* copy numbers by mrcPCR

**Figure 5:**
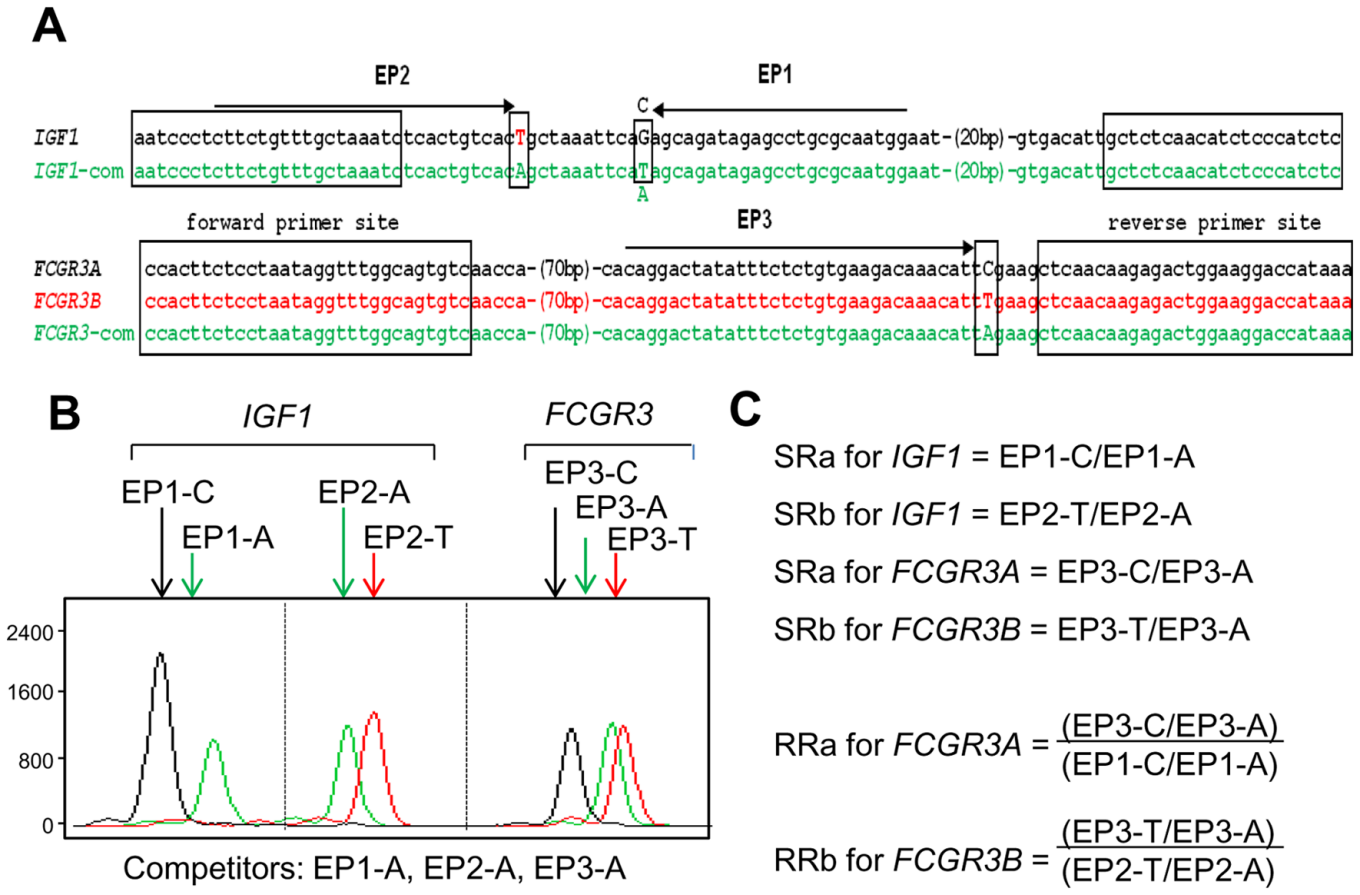
mrcPCR assay for determination of *FCGR3A* and *FCGR3B* copies. (A) Alignment of wild-type and competitor sequences for *IGF1* and *FCGR3* used in mrcPCR assay. The nucleotides corresponding to modified bases are indicated in upper-case letters. The forward and reverse PCR primer sites are marked in brackets. EP1, EP2, and EP3 represent the extension primer sites, with the direction of the extension indicated by arrows. (B) Electrophoregram of extended products in mrcPCR assay. Extended nucleotides are indicated after the extension primers. Four peaks originated from genomic *IGF1* (EP1-C and EP2-T) and competitor *IGF1* (EP1-A and EP2-A), and the other three peaks originated from genomic *FCGR3A* (EP3-C), genomic *FCGR3B* (EP3-T), and competitor *FCGR3* (EP3-A). (C) Formula for the signal ratios (SRs) and the relative ratios (RRs) from peak heights of electrophoregram.

It has been reported that CNVs are associated with disease susceptibility [32], but their validation for each gene or locus has proved challenging. This has been even more difficult for genes having one or several variants with quite similar sequences. Copy status determination for *FCGR3A* and *FCGR3B* is an example. A lower copy number for *FCGR3B* but not *FCGR3A* has been reported to be associated with a higher risk of systemic lupus erythematosus (SLE) [2], [3]. The PRT method recently has been reported to be useful for copy-number determination of *FCGR3A* and *FCGR3B* with the availability of the dispersed repeat sequence similar to the target gene [23]. However, in addition to PRT, another independent methodology, REDVR, is necessary for determination of the copy-number information of those gene variants. In the present study, instead of two independent methodologies, a single mrcPCR assay was employed. For this, two different bases were changed in the reference gene *IGF1* competitor, as shown in Fig. 5A. The representative result was shown in Fig. 5B, and the SR and RR calculation formulae in Fig. 5C.

**Figure 6:**
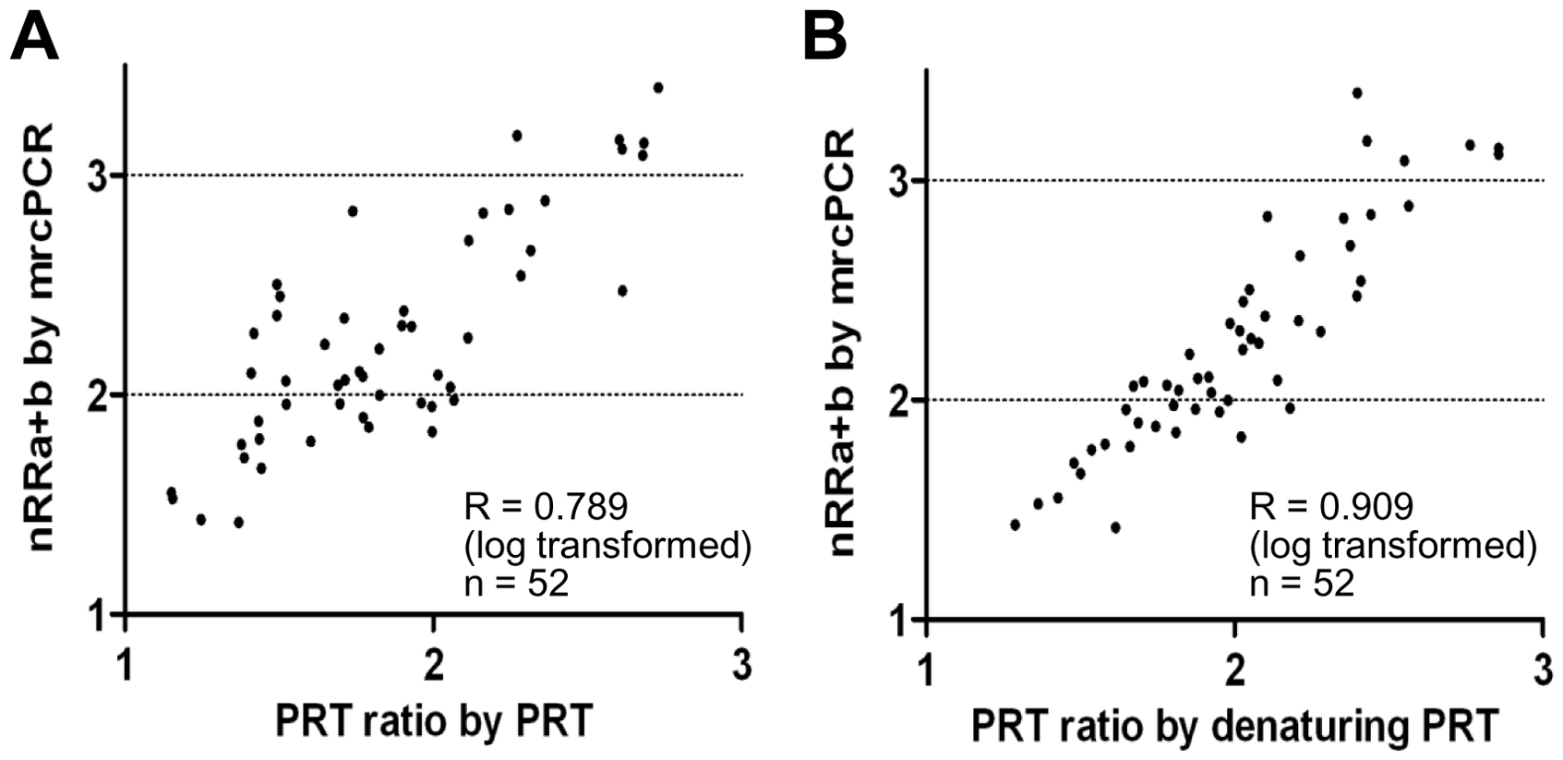
Correlation between the PRT ratio and the nRRa+b according to extra-denaturation procedure. (A) The correlation between the PRT ratio by non-denaturing PRT (PRT without extra-denaturation) and the nRRa+b by mrcPCR (R = 0.789, n = 52). (B) The correlation between the PRT ratio by denaturing PRT (PRT with extra-denaturation) and the nRRa+b by mrcPCR (R = 0.909, n = 52). The PRT ratio is the peak area ratio of *FCGR3* to the reference sequence on chromosome 18 by PRT (x-axis). The nRRa+b is the sum of the nRR values of *FCGR3A* and *FCGR3B* by mrcPCR (y-axis).

In comparing the mrcPCR with PRT using fifty two blood genomic DNA samples, we expected that the sum of the nRR values of *FCGR3A* and *FCGR3B* by mrcPCR (nRRa+b) would show a tight linear correlation with the PRT ratio (the peak area ratio between *FCGR3* and the reference sequence on chromosome 18 by PRT). However, the correlation between the PRT ratio and the nRRa+b was not excellent (R = 0.789 after logarithmic transformation; Fig. 6A), though the nRRa+b values from two independent mrcPCR assays were highly correlated (R = 0.923). This relatively lower correlation might hamper accurate estimation of *FCGR3A* and *FCGR3B* copies and lead, thereby, to erroneous conclusions.

In the mrcPCR assays, the extra-denaturation procedure for the competitor, primers, and genomic DNA was routinely employed to solve the plasmid structural problem and to decrease the assay variation. It may have such effects also in the PRT assay. Even though most three-dimensional DNA structures related to histones would be resolved during DNA purification procedures, supramolecular assembly of the DNA structure resulting from various noncovalent interactions such as hydrogen bonding, electrostatics, and π−π stacking [33] could affect the final PRT results. To test for this possibility, we added an extra-denaturation procedure to the PRT assay (denaturing PRT), as in our mrcPCR assay. Surprisingly, with this extra procedure, the correlation between the PRT ratio and nRRa+b was remarkably improved, as shown in Fig. 6B (R = 0.909 after logarithmic transformation), indicating the importance of the extra-denaturation procedure. This raises the issue of complete denaturation prior to PCR for copy-number quantification to resolve remaining genomic DNA structures which could affect the final result of gene copy. In our experience, freshly prepared DNA or freshly diluted DNA, but not that which had undergone several freeze and thaw cycles, seems to be more prone to such variation with respect to the extra-denaturation procedure.

**Figure 7:**
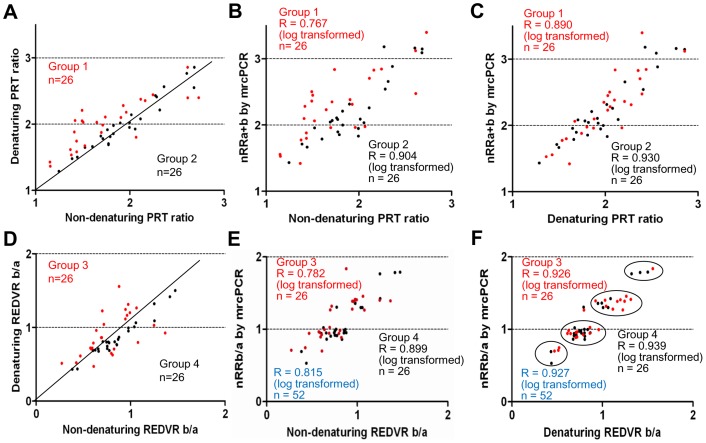
PRT ratios and REDVR b/a according to extra-denaturation procedure. (A) Two groups showing higher (group 1, red) or lower (group 2, black) variation in the PRT ratios according to the extra-denaturation procedure for PRT assays. (B) The correlations of the non-denaturing PRT ratio with the nRRa+b in groups 1 (red) and 2 (black). (C) The correlations of the denaturing PRT ratio with the nRRb/a in groups 1 (red) and 2 (black). (D) Two groups showing higher (group 3, red) and lower (group 4, black) variation in the REDVR b/a according to the extra-denaturation procedure. (E) The correlation of the non-denaturing REDVR b/a with the nRRb/a in groups 3 (red) and 4 (black). The correlation in the total group 3 and 4 cases is shown in blue. (F) The correlation of the denaturing REDVR b/a with the nRRb/a in groups 3 (red) and 4 (black). The correlation in the total group 3 and 4 cases was shown in blue. All correlation-coefficient R values were obtained by Pearson correlation test after logarithmic transformation. PRT ratio, REDVR b/a, nRRa+b, and nRRb/a are defined in Materials and Methods. The non-denaturing PRT ratio and non-denaturing REDVR b/a are the values obtained without the extra-denaturation procedure: the denaturing PRT ratio and denaturing REDVR b/a, with the extra procedure.

For further analysis of the correlation between the PRT and mrcPCR results according to the extra-denaturation procedure, the samples were re-grouped on the basis of the level of variation in the PRT ratios according to the extra-denaturation procedure (the non-denaturing PRT ratio, the PRT ratio by PRT assay without extra-denaturation; the denaturing PRT ratio, the PRT ratio by PRT assay with extra-denaturation): Group 1 is the cases showing higher variation in the PRT ratios between two assays (<0.9 or >1.1; n = 26; red dots in Figs. 7A–7C), and group 2 is the cases showing lower variation (0.919–1.081; n = 26; black dots in Figs. 7A–7C). The correlation between the non-denaturing PRT ratio and nRRa+b was lower in group 1 (R = 0.767 with logarithmic transformation, red dots in Fig. 7B), than in group 2 (R = 0.904 with logarithmic transformation, black dots in Fig. 7B), indicating that the non-denaturing PRT ratio in the cases showing higher variation with the denaturing PRT ratio, also showed higher variation with the mrcPCR result. However, the denaturing PRT ratio in both groups showed similar close correlations with the nRRa+b (R = 0.890 for group 1, red in Fig. 7C; R = 0.930 for group 2 with logarithmic transformation, black in Fig. 7C). These results suggest that the major difference between the non-denaturing PRT and mrcPCR originates in samples showing higher variations according to the extra-denaturation procedure.

In the paper on the development of PRT methodology for *FCGR3A* and *FCGR3B* [23], two PRT assays using two different dyes with the same primer sequences were recommended for each sample, due to the PRT ratio variations in some samples; however, the cause of such variations was not clearly explained. Our data suggest that these can be reduced by the extra-denaturation procedure. The importance of pre-PCR genomic DNA denaturation for molecular copy estimation has not been well recognized. However, our data suggest that the extra-denaturation procedure prior to PCR amplification might be necessary to reduce the assay variation for gene copy determination.

The REDVR b/a (the peak area ratios between 185bp *FCGR3B* and 136bp *FCGR3A*) were compared with the nRRb/a (the ratio of *FCGR3B* and *FCGR3A* nRR values by mrcPCR, Figs. 7D–7F). Similarly to the PRT assay, the denaturing REDVR b/a (the REDVR b/a by REDVR assay with extra-denaturation procedure) showed higher correlation with the nRRb/a (R = 0.927 after logarithmic transformation, Fig. 7F) than the non-denaturing REDVR b/a (the REDVR b/a by REDVR assay without extra-denaturation; R = 0.815 after logarithmic transformation, Fig. 7E). For further analysis, the samples were re-grouped according to the level of variation in the REDVR b/a according to the extra-denaturation procedure, as shown in Fig. 7D. Group 3 is the samples showing higher variation (<0.85, or >1.15; red dots in Figs. 7D–7F), and group 4 is those showing lower variation (0.85–1.15; black dots in Figs. 7D–7F). The correlation of the non-denaturing REDVR b/a with the nRRb/a was lower in group 3 (R = 0. 782 with logarithmic transformation; red dots in Fig. 7E), than that in group 4 (R = 0.899 with logarithmic transformation; black dots in Fig. 7E). By contrast, the denaturing REDVR b/a in both groups showed similar correlations with the nRRb/a (R = 0.926 for group 3; R = 0.939 for group 4) as shown in Fig. 7F. These results suggest that the main difference between the non-denaturing REDVR and the mrcPCR results was the samples showing higher-variation in the REDVR b/a according to the extra-denaturation procedure.

**Figure 8:**
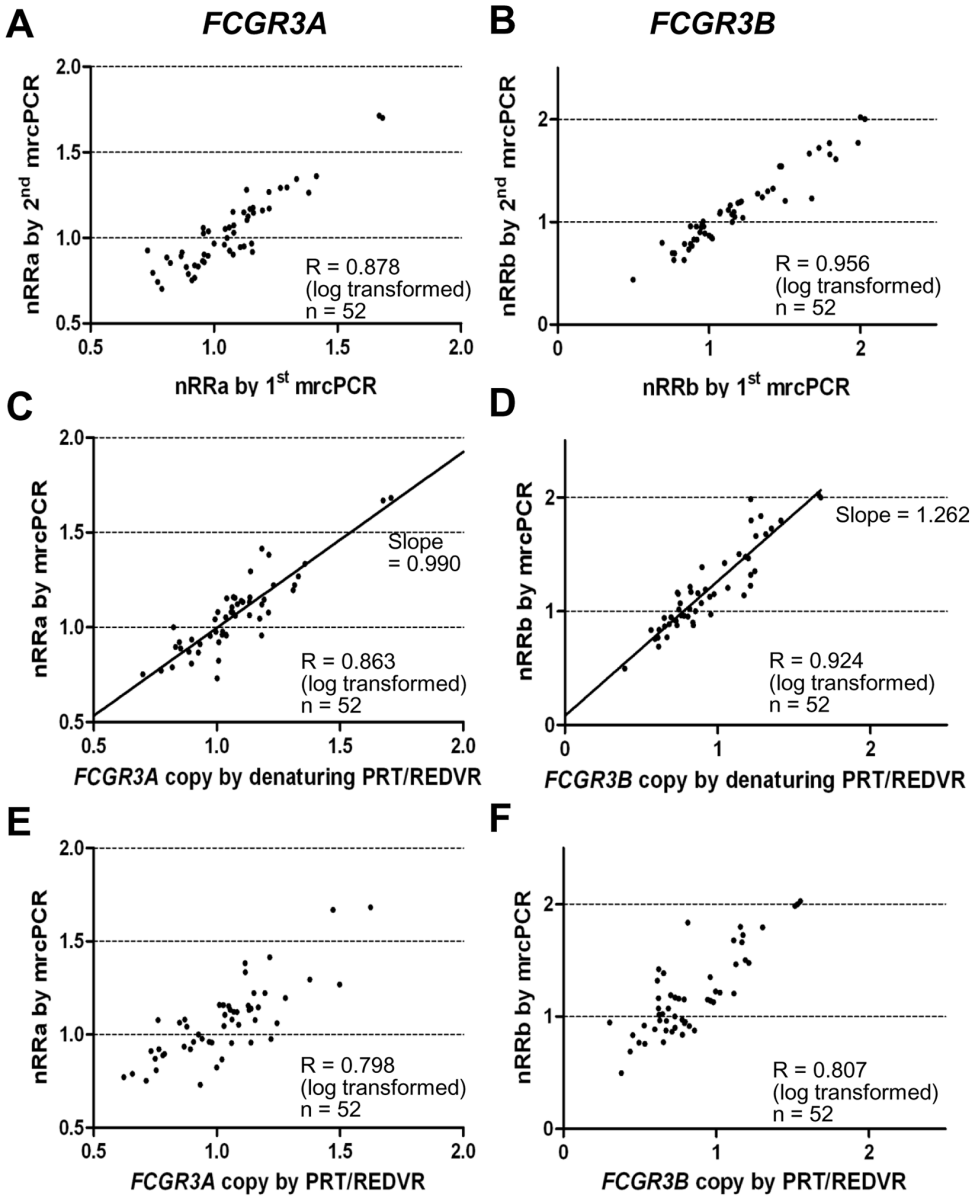
Comparison of *FCGR3A* or *FCGR3B* copies by mrcPCR with those by combined PRT/REDVR methods. The correlation of *FCGR3A* (A) or *FCGR3B* (B) copies by two independent mrcPCR assays. The correlation of *FCGR3A* (C) or *FCGR3B* (D) copies by mrcPCR with those by the combination of denaturing PRT and denaturing REDVR (denaturing PRT/REDVR) methods. The correlation of *FCGR3A* (E) or *FCGR3B* (F) by non-denaturing PRT/REDVR (PRT/REDVR) with those by mrcPCR. All correlation-coefficient R values were obtained by Pearson correlation test after logarithmic transformation. nRRa is the nRR value for *FCGR3A* by mrcPCR; nRRb, for *FCGR3B*. *FCGR3A* and *FCGR3B* copies by PRT/REDVR in the figures are the copies not normalized by median values.

The *FCGR3A* and *FCGR3B* copies by two independent mrcPCR assays showed high correlations (R = 0.878 for *FCGR3A* with log transformation, Fig. 8A; R = 0.956 for *FCGR3B* with log transformation, Fig. 8B), suggesting the reproducibility of mrcPCR assay. In addition, the *FCGR3A* and *FCGR3B* copies by mrcPCR showed high correlations with those by the combined PRT/REDVR methods with the extra-denaturation procedure (R = 0.863 for *FCGR3A* with log transformation, Fig. 8C; R = 0.924 for *FCGR3B* with log transformation, Fig. 8D). By contrast, when the copies by mrcPCR were compared with those by the combined PRT/REDVR methods without the extra-denaturation procedure, the correlation was much lower (R = 0.798 for *FCGR3A* with log transformation, Fig. 8E; R = 0.807 for *FCGR3B* with log transformation, Fig. 8F). These data suggest again the importance of the extra-denaturation procedure.

In comparison of the *FCGR3A* and *FCGR3B* copies by mrcPCR with those by PRT/REDVR, the *FCGR3A* copy numbers were similar as in Fig. 8C, and the slope of the correlation for *FCGR3A* was near 1.0 (slope  = 0.990 with 95% confidence interval of 0.966–1.015). However, the *FCGR3B* copies were different between the mrcPCR and the combined PRT/REDVR methods as in Fig. 8D, and the slope of the correlation for *FCGR3B* was not near 1.0 (slope  = 1.262 with 95% CI of 1.220–1.305). Even with a high correlation between different methodologies, raw data may not be interchangeable before the normalization with standard samples. In the mrcPCR, *FCGR3A* and *FCGR3B* copies have already been corrected by the median values of tested samples as described in Materials and Methods. However, the copy results by PRT/REDVR were not corrected. So, the median value of *FCGR3B* copies by PRT/REDVR from samples was used for the correction as for the copies by mrcPCR. With the corrected *FCGR3B* copy values, the slope of the correlation between the mrcPCR and the combined PRT/REDVR results became 1.011 (95% CI, 0.977–1.045), suggesting that the corrected *FCGR3B* copies using median value could now be interchangeable with those by mrcPCR.

With the extra-denaturation procedure, the PRT ratio showed a high correlation with the nRRa+b (R = 0.904, Fig. 6B). The REDVR b/a also showed a high correlation with the nRRb/a (R = 0.927, Fig. 7E). In addition, the comparison of *FCGR3A* and *FCGR3B* copies by mrcPCR with those by calculated from the combined PRT/REDVR methods showed high correlation (R = 0.863 and 0.924 for *FCGR3A* and *FCGR3B* with log transformation, Figs. 8C–8D). Moreover, the corrected copies for *FCGR3A* and *FCGR3B* were highly consistent between mrcPCR and PRT/REDVR methodologies. These results indicate that mrcPCR results are highly consistent with those from PRT and/or REDVR method. The correlation between the REDVR and mrcPCR results was especially remarkable in that four distinctly separate groups were apparent (marked in circles in Fig. 7F), supporting again that the mrcPCR results are highly consistent with those by PRT/REDVR with the extra-denaturation procedure.

For deduction of the absolute *FCGR3A* and *FCGR3B* copies, we employed the median nRR values from our study population, because the reference genomic DNA samples with known copies were not available. When the copy number of the reference gene is known in study population samples, however, cloned plasmids of target and reference genes can be employed in mrcPCR to estimate the absolute copy numbers. As substituted for genomic DNA, the target and reference plasmids admixed in various ratios were competitively co-amplified with competitors, and the resulting RR values were plotted against the relative ratios between the input amounts of the target and reference plasmids (Figs. S2A and S2B). The RR value for the input ratio of 1.0 between the target and reference plasmids could be the nRR value for two copies of target gene when there are two copies of reference gene per cell. The deduced RR values for two copies of *FCGR3A* and *FCGR3B* were 0.49 and 0.92, quite similar to median nRR values, 0.48 and 0.87, from our study population. The absolute copy values from the plasmid mixtures were also quite similar to those deduced from our study population. These results suggest that in determinations of the absolute copy numbers of the target genes in test samples, employment of target and reference plasmids can be an effective alternative, when reference samples with known copies are unavailable.

In the present study, we showed that copy numbers measured by mrcPCR are highly consistent with the standard or the most sensitive method currently available. In addition, mrcPCR requires as little as 10 ng DNA and about three hours of assay time, so it could be competitive with other currently available molecular methods for measuring copy numbers, including multiplex-ligation-dependent probe amplification (MLPA) [34], multiplex amplification and probe hybridization (MAPH) [35], and quantitative multiplex PCR of short fluorescent fragment (QMPSF) [36]. Furthermore, mrcPCR promises to be an invaluable method for measurement of copy-number variation in genes with variants of similar structures, including *FCGR3* [23], *CCL3L1* [5], and *C4* [37], owing to the facts that, unlike the PRT method, extra-methodology such as REDVR is not necessary [7], [23], and that more versatile application to many genes without dispersed repeat sequences similar to the target gene is possible.

The mrcPCR method also has limitations. It takes an additional approximately two weeks for cloning of competitors, and requires extra-analysis procedures after PCR. Also, different competitor preparations, and different batches of PCR primers or extension primers, might yield inter-assay variations. And as these factors also can interfere with raw-data exchange among independent laboratories, we would recommend using nRR instead of RR for that purpose. For every new batch of primers or competitors, the use of new standard RR values from control samples would be advisable so as to remove any possible assay variation. Additionally, the results of commercially available reference genomic DNA samples can help independent researchers to exchange raw data based on nRR values from those reference samples, and so we included the data on commercially available samples that were used for determination of *C4* copies [37], as shown in Table S7, with SR, RR, and nRR values derived from the signal peaks of *FCGR3A*, *FCGR3B* and *IGF1*. We hope that this effort will facilitate the reproduction of our results and method in independent laboratories, to the greater end that is the development of new, simpler, more reproducible assays.

In the present study, we introduced a modified real competitive PCR (mrcPCR) method employing cloned competitors and automatic sequencer to measure copy-number variations (CNVs) and alterations (CNAs). By comparison of the mrcPCR results with currently available standard or sensitive methods, we demonstrated its consistency, reliability, and versatility with as little as 10 ng genomic DNA and less than three hours of assay time. We firmly believe that this mrcPCR method has great potential as a means of validating candidate CNVs in large case-control association studies and applying clinically relevant CNAs to the treatment of cancer patients.

## Supporting Information

Figure S1 **Comparison of** ***FGFR1*** **copy status between mrcPCR and real-time PCR.**
(DOCX)

Figure S2 **Determination of absolute copy numbers of** ***FCGR3A*** **and** ***FCGR3B*** **with cloned target and reference genes.**
(DOCX)

Table S1 **24 drug-target genes for determination of copy number by mrcPCR.**
(DOCX)

Table S2 **Oligonucleotide primers for determination of** ***ERBB2*** **copy-number status.**
(DOCX)

Table S3 **Oligonucleotide primers for determination of copy-number status of 24 different genes by mrcPCR.**
(DOCX)

Table S4 **Oligonucleotide primers for** ***FCGR3A*** **and** ***FCGR3B*** **by mrcPCR.**
(DOCX)

Table S5 **Oligonucleotide primers for quantitative real-time PCR.**
(DOCX)

Table S6 **Oligonucleotide primers for PRT and REDVR.**
(DOCX)

Table S7 **Results of reference DNA samples by mrcPCR (IHWG consanguineous panel).**
(DOCX)

Methods S1 **Detailed mrcPCR procedure.**
(DOCX)
