# Supplementary material for: Simple and Versatile Molecular Method of Copy-Number Measurement Using Cloned Competitors
Source: PLoS One. 2013 Jul 30;8(7):e69414. doi: 10.1371/journal.pone.0069414 (PMC3728337; doi:10.1371/journal.pone.0069414)
Supplement: Figure S1 — Comparison of FGFR1 copy status between mrcPCR and real-time PCR. (DOCX) [file pone.0069414.s001.docx]

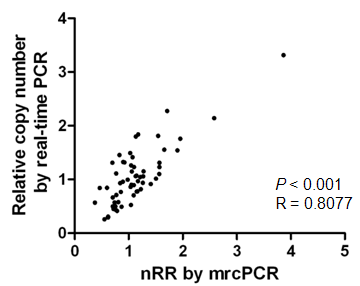


Figure S1. Comparison of *FGFR1* copy status between mrcPCR and real-time PCR. The Pearson correlation coefficients (R) and *P* values after logarithmic transformation are shown.
