# Supplementary material for: Simple and Versatile Molecular Method of Copy-Number Measurement Using Cloned Competitors
Source: PLoS One. 2013 Jul 30;8(7):e69414. doi: 10.1371/journal.pone.0069414 (PMC3728337; doi:10.1371/journal.pone.0069414)
Supplement: Figure S2 — Determination of absolute copy numbers of FCGR3A and FCGR3B with cloned target and reference genes. (DOCX) [file pone.0069414.s002.docx]

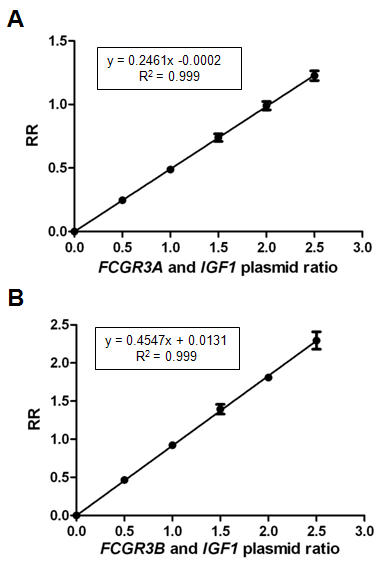


Figure S2. Determination of absolute copy numbers of *FCGR3A* and *FCGR3B* with cloned target and reference genes. A. Determination of absolute *FCGR3A* copies. Various amounts of *FCGR3A* plasmid were admixed with fixed amounts of *FCGR3B* and *IGF1* plasmids. The diluted plasmid mixture was used in the mrcPCR in place of genomic DNA. The RR value of 1.0 which is obtained from the 1:1 ratio between the input amounts of the *FCGR3A* and *IGF1* plasmids was the nRR value for two copies of *FCGR3A* per cell. X-axis: Input ratio between *FCGR3A* and *IGF1* plasmids. Y-axis: RR values for *FCGR3A* plasmid relative to *IGF1* plasmid. B. Determination of absolute *FCGR3B* copies. Various amounts of *FCGR3B* plasmid were admixed with fixed amounts of *FCGR3A* and *IGF1* plasmids. The RR value of 1.0 which is obtained from the 1:1 ratio between the input amounts of the *FCGR3B* and *IGF1* plasmids was the nRR value for two copies of *FCGR3B* per cell. X-axis: input ratio between *FCGR3B* and *IGF1* plasmids. Y-axis: RR values for *FCGR3B* plasmid relative to *IGF1* plasmid. Error bars: 95% confidence interval from three replicates.
