# Supplementary material for: Simple and Versatile Molecular Method of Copy-Number Measurement Using Cloned Competitors
Source: PLoS One. 2013 Jul 30;8(7):e69414. doi: 10.1371/journal.pone.0069414 (PMC3728337; doi:10.1371/journal.pone.0069414)
Supplement: Table S1 — 24 drug-target genes for determination of copy number by mrcPCR. (DOCX) [file pone.0069414.s003.docx]

Table S1. 24 drug-target genes for determination of copy number by mrcPCR.

| Gene | Chromosomal location | Drug | Ref |
| --- | --- | --- | --- |
| RAF1 | 3p25 | PLX4720^c^, AZ628^c^, SB590885^c^ | [[1](#_ENREF_1)], [[2](#_ENREF_2)] |
| PIK3CA | 3q26.32 | NVP-BEZ235^b^, GDC0941^b^ | [[1](#_ENREF_1)] |
| KIT | 4q11-q12 | Sorafenib^a^, Sunitinib^a^, | [[1](#_ENREF_1)] |
| KDR | 4q11-q12 | Sorafenib^a^, Sunitinib^a^, | [[1](#_ENREF_1)] |
| PDGFRA | 4q11-q13 | Imatinib^a^, Sorafenib^a^, | [[1](#_ENREF_1)] |
| hTERT | 5p15.33 | BIBR1532^c^ | [[3](#_ENREF_3)] |
| PDGFRB | 5q31-q32 | Sorafenib^a^, Sunitinib^a^, | [[1](#_ENREF_1)] |
| EGFR | 7p12 | Gefitinib^a^, Erlotinib^a^ | [[1](#_ENREF_1)] |
| ABCB1 | 7q21.12 | CP100356^c^ | [[4](#_ENREF_4)] |
| MET | 7q31.2 | PHA-665752^c,^ PF-02341066^b^ | [[1](#_ENREF_1)] |
| FGFR1 | 8p11.2-p11.1 | PD-173074^c^ | [[1](#_ENREF_1)] |
| PTK2 | 8q24.3 | FAK Inhibitor 14^c^, PF-00562271 ^b^, PF-04554878 ^b^, GSK2256098 ^b^ | [[1](#_ENREF_1)], [[5-8](#_ENREF_5)] |
| MYC | 8q24.12-q24.13 | c-Myc Inhibitor I, c-Myc Inhibitor II | [[9](#_ENREF_9),[10](#_ENREF_10)] |
| ERBB3 | 12q13.2 | AV-203^e^ | [[11](#_ENREF_11)] |
| Akt1 | 14q32.33 | MK-2206^b,^ A-443654^c^, AKT inhibitor VIII^b^ | [[1](#_ENREF_1)] |
| MAP2K1 | 15q22.1-q22.33 | PD-0325901^b,^ RDEA119^b^, CI-1040^b^, AZD6244^b^ | [[1](#_ENREF_1)] |
| IGF1R | 15q26.3 | BMS-536924^c^, BMS-754807^b^, Podophyllotoxin^c^ | [[1](#_ENREF_1)], [[12](#_ENREF_12)] |
| MRP1 | 16p13.11 | Reversan^c^ | [[13](#_ENREF_13)] |
| AURKB | 17p13.1 | ZM-447439^c^, | [[1](#_ENREF_1)] |
| ERBB2 | 17q11.2-q12 | Lapatinib^a^, TAK165^c^, GW583340 ^c^ | [[1](#_ENREF_1)], [[14](#_ENREF_14)] |
| TOP2A | 17q21.2 | Doxorubicin^d^, Etoposide^d^ | [[1](#_ENREF_1)] |
| MAP2K2 | 19p13.3 | PD-0325901^b,^ RDEA119^b^, CI-1040^b^, AZD6244^b^ | [[1](#_ENREF_1)] |
| AURKA | 20q13.2-q13.3 | VX-680^b^ | [[1](#_ENREF_1)] |
| TP63* | 3q28 |  | [[15](#_ENREF_15)] |

^a^ Clinical

^b^ In clinical development

^c^ Experimental

^d^ Chemotherapy

^e^ Antibody

*There is currently no inhibitory compound for *TP63* activity, but it is a possible target for anti-cancer drugs.

References

1. Garnett, M.J., Edelman, E.J., Heidorn, S.J., Greenman, C.D., Dastur, A., Lau, K.W., Greninger, P., Thompson, I.R., Luo, X., Soares, J. *et al.* (2012) Systematic identification of genomic markers of drug sensitivity in cancer cells. *Nature*, **483**, 570-575.

2. Hatzivassiliou, G., Song, K., Yen, I., Brandhuber, B.J., Anderson, D.J., Alvarado, R., Ludlam, M.J., Stokoe, D., Gloor, S.L., Vigers, G. *et al.* RAF inhibitors prime wild-type RAF to activate the MAPK pathway and enhance growth. *Nature*, **464**, 431-435.

3. Damm, K., Hemmann, U., Garin-Chesa, P., Hauel, N., Kauffmann, I., Priepke, H., Niestroj, C., Daiber, C., Enenkel, B., Guilliard, B. *et al.* (2001) A highly selective telomerase inhibitor limiting human cancer cell proliferation. *EMBO J*, **20**, 6958-6968.

4. Wandel, C., Kim, R.B., Kajiji, S., Guengerich, P., Wilkinson, G.R. and Wood, A.J. (1999) P-glycoprotein and cytochrome P-450 3A inhibition: dissociation of inhibitory potencies. *Cancer Res*, **59**, 3944-3948.

5. Molckovsky, A. and Siu, L.L. (2008) First-in-class, first-in-human phase I results of targeted agents: highlights of the 2008 American society of clinical oncology meeting. *J Hematol Oncol*, **1**, 20.

6. Siu, L.L., Burris, H.A., Mileshkin, L., Camidge, D.R., Rischin, D., Chen, E.X., Jones, S., Yin, D. and Fingert, H. (2007) Phase 1 study of a focal adhesion kinase (FAK) inhibitor PF-00562271 in patients (pts) with advanced solid tumors. *J Clin Oncol, ASCO Annual Meeting Proceedings (Post-Meeting Edition),* , **25** 18S (June 20 Supplement, abstract # 3527).

7. Jones, S.F., Shapiro, G., Bendell, J.C., Chen, E.X., Bedard, P., Cleary, J.M., Pandya, S., Pierce, K.J., Houk, B., Hosea, N. *et al.* (2011) Phase I study of PF-04554878, a second-generation focal adhesion kinase (FAK) inhibitor, in patients with advanced solid tumors. *J Clin Oncol* **29**, (suppl; abstr 3002).

8. Soria, J.-C., Gan, H.K., Arkenau, H.-T., Blagden, S.P., Plummer, R., Ranson, M., Jeffry Evans, T.R., Zalcman, G., Bahleda, R., Hollebecque, A. *et al.* (2012) Phase I clinical and pharmacologic study of the focal adhesion kinase (FAK) inhibitor GSK2256098 in pts with advanced solid tumors. *J Clin Oncol* **30**, (suppl; abstr 3000)

9. Huang, M.J., Cheng, Y.C., Liu, C.R., Lin, S. and Liu, H.E. (2006) A small-molecule c-Myc inhibitor, 10058-F4, induces cell-cycle arrest, apoptosis, and myeloid differentiation of human acute myeloid leukemia. *Exp Hematol*, **34**, 1480-1489.

10. Clausen, D.M., Guo, J., Parise, R.A., Beumer, J.H., Egorin, M.J., Lazo, J.S., Prochownik, E.V. and Eiseman, J.L. (2010) In vitro cytotoxicity and in vivo efficacy, pharmacokinetics, and metabolism of 10074-G5, a novel small-molecule inhibitor of c-Myc/Max dimerization. *J Pharmacol Exp Ther*, **335**, 715-727.

11. Vincent, S., Fleet, C., Bottega, S., McIntosh, D., Winston, W. and Chen, T. (2012) Abstract 2509: AV-203, a humanized ERBB3 inhibitory antibody inhibits ligand-dependent and ligand-independent ERBB3 signaling in vitro and in vivo. *Cancer Research*, **72**, Supplement 1

12. Girnita, A., Girnita, L., del Prete, F., Bartolazzi, A., Larsson, O. and Axelson, M. (2004) Cyclolignans as inhibitors of the insulin-like growth factor-1 receptor and malignant cell growth. *Cancer Res*, **64**, 236-242.

13. Burkhart, C.A., Watt, F., Murray, J., Pajic, M., Prokvolit, A., Xue, C., Flemming, C., Smith, J., Purmal, A., Isachenko, N. *et al.* (2009) Small-molecule multidrug resistance-associated protein 1 inhibitor reversan increases the therapeutic index of chemotherapy in mouse models of neuroblastoma. *Cancer Res*, **69**, 6573-6580.

14. Gaul, M.D., Guo, Y., Affleck, K., Cockerill, G.S., Gilmer, T.M., Griffin, R.J., Guntrip, S., Keith, B.R., Knight, W.B., Mullin, R.J. *et al.* (2003) Discovery and biological evaluation of potent dual ErbB-2/EGFR tyrosine kinase inhibitors: 6-thiazolylquinazolines. *Bioorg Med Chem Lett*, **13**, 637-640.

15. McKeon, F.D. (2004) p63 and p73 in tumor suppression and promotion. *Cancer Res Treat*, **36**, 6-12.
