# Supplementary material for: Simple and Versatile Molecular Method of Copy-Number Measurement Using Cloned Competitors
Source: PLoS One. 2013 Jul 30;8(7):e69414. doi: 10.1371/journal.pone.0069414 (PMC3728337; doi:10.1371/journal.pone.0069414)
Supplement: Table S2 — Oligonucleotide primers for determination of ERBB2 copy-number status. (DOCX) [file pone.0069414.s004.docx]

Table S2. Oligonucleotide primers for determination of *ERBB2* copy-number status.

| Gene | Size | Primers^b^ | Sequence |
| --- | --- | --- | --- |
| *ERBB2*^a^  (*3-ERBB2*) | 117 | Forward primer | TGTCCCCAGGAAGCATACGTGAT |
|  |  | Reverse primer | GCAGCCATAGGGCATAAGCTGTG |
|  | 21 | Extension primer | GGGCATCTGCCTGACATCCAC |
|  |  | Modified sequence^c^ | CTTCTGGGCATCTGCCTGACATCCACgGTG |
| *ERBB2*^a^  (5- *ERBB2*) | 102 | Forward primer | GGGCTACGTGCTCATCGCTCAC |
|  |  | Reverse primer | GCCAGGGCATAGTTGTCCTCAAAG |
|  | 27 | Extension primer | CAAAGAGCTGGGTGCCTCGCACAATCC |
|  |  | Modified sequence^c^ | AGGCTGcGGATTGTGCGAGGCACCCAGCTCTTTGAGG |
| *G6PC3* | 90 | Forward primer | CTCCAGCCCAGGTTCACCAGTTC |
|  |  | Reverse primer | CCTAACCACATTGGGAAAGGGAGAAT |
|  | 31 | Extension primer | GTTCACgAGTTCCCCTCTTCTTGTGAGACTG |
|  |  | Modified sequence^c^ | CCCAGGTTCACCAGTTCCCCTCTTCTTGTGAGACTGgTCC |
| *ALDOC* | 139 | Forward primer | GGGCAGAGTAATGAGGTTGGCACT |
|  |  | Reverse primer | GTGCAGAGGGTGTACGCTCACTGAT |
|  | 35 | Extension primer | cacactctAGCACACAGCGCCACTTGGCAAAGTCA |
|  |  | Modified sequence^c^ | TGGTGcTGACTTTGCCAAGTGGCGCTGTGTGCTGAAA |

^a^The two *ERBB2* sequences used in this study are referenced as 3-*ERBB2* and 5-*ERBB2* in the text.

^b^The primer information for multiplex PCR (forward and reverse primers) and single-base extension (extension primer) along with the sites of the modified bases for competitors sequences (modified sequence) are shown. The extension primers for *G6PC3* and *ALDOC* were modified from the original genomic sequence, and the lowercase characters are the modified bases.

^c^The modified bases for competitors sequences are indicated by lowercase characters in the modified sequence. The sites for extension primers are underlined.
