# Supplementary material for: Simple and Versatile Molecular Method of Copy-Number Measurement Using Cloned Competitors
Source: PLoS One. 2013 Jul 30;8(7):e69414. doi: 10.1371/journal.pone.0069414 (PMC3728337; doi:10.1371/journal.pone.0069414)
Supplement: Table S3 — Oligonucleotide primers for determination of copy-number status of 24 different genes by mrcPCR. (DOCX) [file pone.0069414.s005.docx]

Table S3. Oligonucleotide primers for determination of copy-number status of 24 different genes by mrcPCR.

| PCR | Gene | Size^b^ | Primers^c^ | Sequence |
| --- | --- | --- | --- | --- |
| A^a^ | *ERBB2* | 204 | Forward primer | TGTCCCCAGGAAGCATACGTGAT |
|  |  |  | Reverse primer | CCCAGGTGCATACCTTGGCAAT |
|  |  | 24 | Extension primer | CTTATGCCCTATGGCTGCCTCTTA |
|  |  |  | Modified sequence | CACAGCTTATGCCCTATGGCTGCCTCTTAgACC |
|  | *FGFR1* | 152 | Forward primer | GCATCCATGAACTCTGGGGTTCTTC |
|  |  |  | Reverse primer | CTTCAAAAAGTTGGGAGTCAAAGTATTATTACC |
|  |  | 33 | Extension primer | TTCTGGTTCGGCCATCACGGCTCTCCTCCAGTG |
|  |  |  | Modified sequence | CTTCTGGTTCGGCCATCACGGCTCTCCTCCAGTGgG |
|  | *KIT* | 121 | Forward primer | TTCTTGGCAGGCTCTTCTCAACCA |
|  |  |  | Reverse primer | CAGTGCATAACAGCCTAATCTCGTCG |
|  |  | 30 | Extension primer | GCAGGCTCTTCTCAACCATCTGTGAGTCCA |
|  |  |  | Modified sequence | TTGGCAGGCTCTTCTCAACCATCTGTGAGTCCAgGG |
|  | *AURKA* | 108 | Forward primer | TGGAGCCTTGGAGTTCTTTGCTATG |
|  |  |  | Reverse primer | TCAGTTGCGTCTTACCCGTGATATTC |
|  |  | 27 | Extension primer | ACTCTTGGTATGTGTTTGCCTCAAAAG |
|  |  |  | Modified sequence | AGCCTcCTTTTGAGGCAAACACATACCAAGAGACC |
|  | *IGF1* | 132 | Forward primer | TTTTCTCTAAATCCCTCTTCTGTTTGCTAAATC |
|  |  |  | Reverse primer | GAGAGATGGGAGATGTTGAGAGCAATGT |
|  |  | 22 | Extension primer | TTGCGCAGGCTCTATCTGCTCT |
|  |  |  | Modified sequence | AATTcAGAGCAGATAGAGCCTGCGCAATGGA |
| B^a^ | *MAP2K2* | 223 | Forward primer | CAAAGACGATGACTTCGAAAGGATCTCAGAG |
|  |  |  | Reverse primer | GAGGGACAGAGCCTGGAGCTAATCAG |
|  |  | 19 | Extension primer | GACCCTCGGGCCTCATCAT |
|  |  |  | Modified sequence | GCACAGACCCTCGGGCCTCATCATgGCC |
|  | *MAP2K1* | 181 | Forward primer | AACTCTCCGTACATCGTGGGCTTCTATGGTG |
|  |  |  | Reverse primer | ATaaaAGcAGCAGCAGGGAGGTAGCTGGTCA |
|  |  | 25 | Extension primer | AGATACTGATCTCGCCATCGCTGTA |
|  |  |  | Modified sequence | GCGTTcTACAGCGATGGCGAGATCAGTATCTGCAT |
|  | *KDR* | 123 | Forward primer | GATCTACGTTTGAGAACCTCACATGGTA |
|  |  |  | Reverse primer | GGTGGCATTCAATTTCCAAAGAGTATC |
|  |  | 26 | Extension primer | TCCaACATGGATTGGCAGAGGCTGTG |
|  |  |  | Modified sequence | TTGGCcCACAGCCTCTGCCAATCCATGTtGGAGAG |
|  | *PDGFRA* | 113 | Forward primer | CCTGCATGAAGTCGAACATTTTGTTGTAGAG |
|  |  |  | Reverse primer | CAGTGGTGATCTCAGTGAGATTTTCAATC |
|  |  | 31 | Extension primer | GTGATCTCAGTGAGATTTTCAATCAGAGTCA |
|  |  |  | Modified sequence | ATcTGACTCTGATTGAAAATCTCACTGAGATCACC |
|  | *IGF1* | 128 | Forward primer | TTCTCTAAATCCCTCTTCTGTTTGCTAAATC |
|  |  |  | Reverse primer | GAGATGGGAGATGTTGAGAGCAATGT |
|  |  | 23 | Extension primer | ATTGCGCAGGCTCTATCTGCTCT |
|  |  |  | Modified sequence | AATTcAGAGCAGATAGAGCCTGCGCAATGGA |
| C^a^ | *AURKB* | 207 | Forward primer | CTGCAGAAGAGCTGCACATTTGAC |
|  |  |  | Reverse primer | GAATCACCTTCTTCCCATGGCAGTAC |
|  |  | 22 | Extension primer | GGCTGAGGGTGTGCTGCTTGTA |
|  |  |  | Modified sequence | CTCGAGGCTGAGGGTGTGCTGCTTGTAgCTG |
|  | *IGF1R* | 180 | Forward primer | CACAGCTCTGCAGTGAACAATTGAAC |
|  |  |  | Reverse primer | TGCTGATAGTCGTTGCGGATGT |
|  |  | 29 | Extension primer | ACGTTTACCCTCTTGTCTCCCTTCAGTCT |
|  |  |  | Modified sequence | GAGACGTTTACCCTCTTGTCTCCCTTCAGTCTgCGG |
|  | *PTK2* | 167 | Forward primer | AGTATGTCCCTATGGTGAAGGTAAAATAGAGTC |
|  |  |  | Reverse primer | CACTGTATGGTGCTTTCGTGAAGAGC |
|  |  | 31 | Extension primer | GCAAAAATGTTCTTGTTTCCTGTAAGCTGTG |
|  |  |  | Modified sequence | TGGCAAAAATGTTCTTGTTTCCTGTAAGCTGTGgTG |
|  | *PDGFRB* | 134 | Forward primer | GTGTTGTATTTTCCCAAGCCCCTTGA |
|  |  |  | Reverse primer | CCTTCACAGATGAGCACGTTCCTAGC |
|  |  | 20 | Extension primer | CCTCTCCCCAAACCCAGTGC |
|  |  |  | Modified sequence | GCTCCCCTCTCCCCAAACCCAGTGCgTCCACAG |
|  | *IGF1* | 128 | Forward primer | TTCTCTAAATCCCTCTTCTGTTTGCTAAATC |
|  |  |  | Reverse primer | GAGATGGGAGATGTTGAGAGCAATGT |
|  |  | 23 | Extension primer | ATTGCGCAGGCTCTATCTGCTCT |
|  |  |  | Modified sequence | AATTcAGAGCAGATAGAGCCTGCGCAATGGA |
| O^a^ | *P63* | 183 | Forward primer | GGACCCCAAGCAGTGCCTCTA |
|  |  |  | Reverse primer | CCTCCTCTTTGATGCGCTGTTG |
|  |  | 34 | Extension primer | ttagagaaGAGTGGAATGACTTCAACTTTGACAT |
|  |  |  | Modified sequence | GAGATGAGTGGAATGACTTCAACTTTGACATgGAT |
|  | *MET* | 157 | Forward primer | GAATGTGAATATGAAGTATCAGCTTCCCAAC |
|  |  |  | Reverse primer | CCCAGTCTTGTACTCAGCAACCTTCTG |
|  |  | 32 | Extension primer | TTAGTGGCACCAAGGAAAATGTGtTGCTCAT |
|  |  |  | Modified sequence | CTcATGAGCATCACATTTTCCTTGGTGCCACTAACT |
|  | *MYC* | 122 | Forward primer | GGCTCCTGGCAAAAGGTCAGAGTCT |
|  |  |  | Reverse primer | GCGCTGCGTAGTTGTGCTGATGT |
|  |  | 28 | Extension primer | AACCTCCTCACAGCCCACTGGTCCTCAA |
|  |  |  | Modified sequence | GCAAACCTCCTCACAGCCCACTGGTCCTCAAgAGG |
|  | *hTERT* | 112 | Forward primer | TGTCCGCCATCCTCTCAGGTTTC |
|  |  |  | Reverse primer | GAGGGAGGCCGTGTCAGAGATGA |
|  |  | 25 | Extension primer | GTGTGCTGCAGCTCCCATTTCATCA |
|  |  |  | Modified sequence | CATGTGTGCTGCAGCTCCCATTTCATCAgCAAGTTT |
|  | *IGF1* | 128 | Forward primer | TTCTCTAAATCCCTCTTCTGTTTGCTAAATC |
|  |  |  | Reverse primer | GAGATGGGAGATGTTGAGAGCAATGT |
|  |  | 22 | Extension primer | TTGCGCAGGCTCTATCTGCTCT |
|  |  |  | Modified sequence | AATTcAGAGCAGATAGAGCCTGCGCAATGGA |
| H^a^ | *MRP1* | 166 | Forward primer | ACATGAAGAGCAAAGACAATCGGATCA |
|  |  |  | Reverse primer | CCCACGGCTGACAGGTAGGCAGACT |
|  |  | 20 | Extension primer | CCTGGGAGCTGGCATTCAAG |
|  |  |  | Modified sequence | TTATGCCTGGGAGCTGGCATTCAAGgACAAGGTGC |
|  | *EGFR* | 150 | Forward primer | GAGGTGGCTGGTTATGTCCTCATTG |
|  |  |  | Reverse primer | CTTCAGTCCGGTTTTATTTGCATCAT |
|  |  | 33 | Extension primer | GCATCATAGTTAGATAAGACTGCTAAGGCATAG |
|  |  |  | Modified sequence | TcCTATGCCTTAGCAGTCTTATCTAACTATGATGCA |
|  | *TOP2A* | 137 | Forward primer | CTTTAGCTCTTTGGCTCGATTGTTATTTC |
|  |  |  | Reverse primer | CTTCAGCACCATTTATCAGCACCAT |
|  |  | 26 | Extension primer | TTTTATATGATGACAACCAGCGTGTTGA |
|  |  |  | Modified sequence | AGTTTTTATATGATGACAACCAGCGTGTTGAgCCTG |
|  | *PIK3CA* | 113 | Forward primer | CTCCACGACCATCATCAGGTGAAC |
|  |  |  | Reverse primer | CTCACGGAGGCATTCTAAAGTCACTATCA |
|  |  | 31 | Extension primer | CATTCTAAAGTCACTATCATTCCATTTGGTA |
|  |  |  | Modified sequence | TAcTACCAAATGGAATGATAGTGACTTTAGAATGC |
|  | *IGF1* | 128 | Forward primer | TTCTCTAAATCCCTCTTCTGTTTGCTAAATC |
|  |  |  | Reverse primer | GAGATGGGAGATGTTGAGAGCAATGT |
|  |  | 25 | Extension primer | CCATTGCGCAGGCTCTATCTGCTCT |
|  |  |  | Modified sequence | AATTcAGAGCAGATAGAGCCTGCGCAATGGA |
| H3^a^ | *HER3* | 163 | Forward primer | GCACCCTTTCTTCAGTGGGTCTC |
|  |  |  | Reverse primer | GACCCCACATCCATGTACTCATAACC |
|  |  | 31 | Extension primer | TTCAGTGGGTCTCAGTTCTGTCCTGGGTACT |
|  |  |  | Modified sequence | TCTTCAGTGGGTCTCAGTTCTGTCCTGGGTACTgAA |
|  | *ABCB1* | 145 | Forward primer | CAGTCATCTGTGGTGAGGCTGATTG |
|  |  |  | Reverse primer | CAGCGGCCTCTGCTTCTTTGAG |
|  |  | 20 | Extension primer | TGATTGGCTGGGCAGGAACA |
|  |  |  | Modified sequence | GGCTGATTGGCTGGGCAGGAACAgCGCC |
|  | *AKT1* | 112 | Forward primer | CCACCCTTCAAGCCCCAGGTCAC |
|  |  |  | Reverse primer | GGCAGTGGCCTCTCACCTTGGTCAG |
|  |  | 36 | Extension primer | GATCATCTGtGCCGTGAACTCCTCATCAAAATACCT |
|  |  |  | Modified sequence | CACcAGGTATTTTGATGAGGAGTTCACGGCcCAGATGATCACC |
|  | *RAF1* | 88 | Forward primer | AAAGCACGCTTAGATTGGAATACTGATG |
|  |  |  | Reverse primer | GTGTTGTGAGGGGAACATGATCCA |
|  |  | 26 | Extension primer | GAATACTGATGCTGCGTCTTTGATTG |
|  |  |  | Modified sequence | TTGGAATACTGATGCTGCGTCTTTGATTGgAGAAG |
|  | *IGF1* | 128 | Forward primer | TTCTCTAAATCCCTCTTCTGTTTGCTAAATC |
|  |  |  | Reverse primer | GAGATGGGAGATGTTGAGAGCAATGT |
|  |  | 25 | Extension primer | CCATTGCGCAGGCTCTATCTGCTCT |
|  |  |  | Modified sequence | AATTcAGAGCAGATAGAGCCTGCGCAATGGA |

^a^Six different multiplex PCRs (A, B, C, O, H, and H3) were employed.

^b^The base lengths of the PCR products or extension primers are shown.

^c^The primers for each multiplex PCR (forward and reverse primers) and single-base extension (extension primer) along with the sites of modified bases for competitors sequences (modified sequence) are shown. The lowercase characters in the primers are the modified base. The modified bases for the competitors sequences are indicated by lowercase characters in the modified sequence. The sites for extension primers are underlined.
