# Supplementary material for: Simple and Versatile Molecular Method of Copy-Number Measurement Using Cloned Competitors
Source: PLoS One. 2013 Jul 30;8(7):e69414. doi: 10.1371/journal.pone.0069414 (PMC3728337; doi:10.1371/journal.pone.0069414)
Supplement: Table S4 — Oligonucleotide primers for FCGR3A and FCGR3B by mrcPCR. (DOCX) [file pone.0069414.s006.docx]

Table S4. Oligonucleotide primers for *FCGR3A* and *FCGR3B* by mrcPCR.

| Gene | Size | Primers | Sequence |
| --- | --- | --- | --- |
| *IGF1* | 132 | FP (Forward primer) | TTCTCTAAATCCCTCTTCTGTTTGCTAAATC |
|  |  | RP (Reverse primer) | GAGATGGGAGATGTTGAGAGCAATGT |
|  | 23 | EP1 (extension primer) | CCATTGCGCAGGCTCTATCTGCT |
|  | 27 | EP2 (extension primer) | CTTCTGTTTGCTAAATCTCACTGTCAC |
| *FCGR3* | 171 | FP (Forward primer) | CCACTTCTCCTAATAAGGTTTGGCAGTGTC |
|  |  | RP (Reverse primer) | TTTATGGTCCTTCCAGTCTCTTGTTGAG |
|  | 32 | EP3 (extension primer) | CAGGACTATATTTCTCTGTGAAGACAAACATT |
