# Supplementary material for: Simple and Versatile Molecular Method of Copy-Number Measurement Using Cloned Competitors
Source: PLoS One. 2013 Jul 30;8(7):e69414. doi: 10.1371/journal.pone.0069414 (PMC3728337; doi:10.1371/journal.pone.0069414)
Supplement: Table S5 — Oligonucleotide primers for quantitative real-time PCR. (DOCX) [file pone.0069414.s007.docx]

Table S5. Oligonucleotide primers for quantitative real-time PCR.

| Gene | Size | Primers | Sequence |
| --- | --- | --- | --- |
| *PTK2* | 301 | Forward primer | CTGAACTATATCCCGCACACAC |
|  |  | Reverse primer | CTTCACCATAGGGACATACTCCTC |
| *MYC* | 319 | Forward primer | CAGGCTTAGATGTGGCTCTTTG |
|  |  | Reverse primer | GTAGTTGTGCTGATGTGTGGAGAC |
| *IGF1* | 302 | Forward primer | CAGCAGTCTTCCAACCCAAT |
|  |  | Reverse primer | CCTGCAGAAGTGGAGGATTTAG |
