# Supplementary material for: Simple and Versatile Molecular Method of Copy-Number Measurement Using Cloned Competitors
Source: PLoS One. 2013 Jul 30;8(7):e69414. doi: 10.1371/journal.pone.0069414 (PMC3728337; doi:10.1371/journal.pone.0069414)
Supplement: Table S6 — Oligonucleotide primers for PRT and REDVR. (DOCX) [file pone.0069414.s008.docx]

Table S6. Oligonucleotide primers for PRT and REDVR.

| Gene | Size | Primers | Sequence |
| --- | --- | --- | --- |
| PRT | 75 | Forward primer | FAM-TGCCCTTCATGATCTGGCCCTGAAAC |
|  |  | Reverse primer | TGAGTTCAAGAAAGCAGTTTGGTTCTG |
| REDVR | 185 | Forward primer | CCTTTTTGCAGTGGACACAGGACTAT |
|  |  | Reverse primer | HEX-GGGTTGCAAATCCAGAGAAATGT |
