# Supplementary material for: Simple and Versatile Molecular Method of Copy-Number Measurement Using Cloned Competitors
Source: PLoS One. 2013 Jul 30;8(7):e69414. doi: 10.1371/journal.pone.0069414 (PMC3728337; doi:10.1371/journal.pone.0069414)
Supplement: Table S7 — Results of reference DNA samples by mrcPCR (IHWG consanguineous panel). (DOCX) [file pone.0069414.s009.docx]

Table S7. Results of reference DNA samples by mrcPCR (IHWG consanguineous panel).

| Samples | SRa for IGF1 | SRb for IGF1 | SRa for  FCGR3A | SRb for  FCGR3B | RRa for  FCGR3A | RRb for  FCGR3B | nRR for  FCGR3A | nRR for  FCGR3B |
| --- | --- | --- | --- | --- | --- | --- | --- | --- |
| IHW09004 | 2.871 | 1.469 | 1.525 | 1.765 | 0.531 | 1.202 | 1.030 | 1.239 |
| IHW09005 | 2.645 | 1.398 | 1.319 | 1.303 | 0.499 | 0.932 | 0.967 | 0.960 |
| IHW09006 | 2.724 | 1.376 | 1.483 | 1.365 | 0.545 | 0.992 | 1.056 | 1.022 |
| IHW09010 | 2.784 | 1.424 | 1.434 | 1.359 | 0.515 | 0.954 | 0.999 | 0.983 |
| IHW09015 | 2.676 | 1.360 | 0.898 | 1.236 | 0.335 | 0.909 | 0.651 | 0.937 |
| IHW09016 | 3.093 | 1.591 | 1.629 | 1.545 | 0.527 | 0.971 | 1.021 | 1.001 |
| IHW09017 | 2.856 | 1.436 | 1.287 | 1.168 | 0.451 | 0.813 | 0.874 | 0.838 |
| IHW09020 | 2.955 | 1.458 | 1.523 | 1.419 | 0.515 | 0.973 | 0.999 | 1.003 |
| IHW09022 | 2.484 | 1.314 | 1.270 | 1.200 | 0.511 | 0.913 | 0.992 | 0.942 |
| IHW09023 | 2.787 | 1.471 | 1.668 | 1.591 | 0.598 | 1.081 | 1.160 | 1.114 |
| IHW09029 | 3.098 | 1.555 | 1.736 | 1.043 | 0.560 | 0.671 | 1.087 | 0.691 |
| IHW09030 | 2.927 | 1.517 | 2.069 | 0.979 | 0.707 | 0.645 | 1.371 | 0.665 |
| IHW09031 | 2.908 | 1.554 | 1.474 | 1.627 | 0.507 | 1.047 | 0.983 | 1.079 |
| IHW09032 | 2.928 | 1.470 | 1.408 | 1.345 | 0.481 | 0.915 | 0.932 | 0.943 |
| IHW09033 | 2.928 | 1.598 | 1.719 | 1.682 | 0.587 | 1.052 | 1.139 | 1.085 |
| IHW09034 | 2.779 | 1.581 | 1.598 | 1.575 | 0.575 | 0.996 | 1.115 | 1.026 |
| IHW09035 | 2.693 | 1.469 | 1.452 | 1.431 | 0.539 | 0.975 | 1.045 | 1.005 |
| IHW09036 | 2.478 | 1.276 | 1.192 | 1.108 | 0.481 | 0.868 | 0.933 | 0.895 |
| IHW09039 | 3.043 | 1.499 | 1.666 | 1.497 | 0.547 | 0.999 | 1.061 | 1.030 |
| IHW09040 | 2.711 | 1.351 | 1.496 | 1.259 | 0.552 | 0.932 | 1.070 | 0.961 |
| IHW09042 | 2.828 | 1.450 | 1.570 | 1.465 | 0.555 | 1.011 | 1.077 | 1.042 |
| IHW09043 | 3.079 | 1.546 | 1.877 | 1.723 | 0.609 | 1.115 | 1.182 | 1.149 |
| IHW09044 | 3.079 | 1.577 | 1.731 | 1.091 | 0.562 | 0.692 | 1.090 | 0.713 |
| IHW09047 | 3.068 | 1.546 | 1.770 | 1.640 | 0.577 | 1.060 | 1.119 | 1.093 |
| IHW09048 | 2.827 | 1.447 | 1.557 | 1.521 | 0.551 | 1.051 | 1.068 | 1.084 |
| IHW09049 | 2.469 | 1.319 | 1.049 | 1.045 | 0.425 | 0.793 | 0.823 | 0.817 |
| IHW09050 | 2.579 | 1.366 | 1.108 | 1.157 | 0.430 | 0.847 | 0.833 | 0.873 |
| IHW09051 | 2.831 | 1.489 | 1.429 | 1.403 | 0.505 | 0.942 | 0.979 | 0.971 |
| IHW09052 | 3.141 | 1.578 | 1.694 | 2.116 | 0.539 | 1.341 | 1.045 | 1.382 |
| IHW09060 | 2.916 | 1.437 | 1.462 | 1.384 | 0.501 | 0.963 | 0.972 | 0.992 |
| IHW09061 | 2.951 | 1.513 | 1.552 | 1.932 | 0.526 | 1.277 | 1.020 | 1.316 |
| IHW09062 | 2.536 | 1.345 | 1.180 | 1.186 | 0.465 | 0.882 | 0.902 | 0.909 |
| IHW09063 | 2.676 | 1.386 | 1.433 | 1.422 | 0.535 | 1.026 | 1.038 | 1.057 |
| IHW09064 | 2.710 | 1.414 | 1.376 | 1.329 | 0.508 | 0.940 | 0.985 | 0.969 |
| IHW09065 | 2.361 | 1.247 | 1.016 | 1.249 | 0.430 | 1.002 | 0.834 | 1.033 |
| IHW09068 | 2.331 | 1.172 | 0.713 | 0.710 | 0.306 | 0.606 | 0.593 | 0.624 |
| IHW09069 | 2.550 | 1.257 | 1.149 | 1.069 | 0.451 | 0.850 | 0.874 | 0.876 |
| IHW09072 | 2.834 | 1.444 | 1.489 | 1.416 | 0.525 | 0.981 | 1.019 | 1.011 |
| IHW09075 | 2.685 | 1.417 | 1.319 | 0.922 | 0.491 | 0.651 | 0.953 | 0.671 |
| IHW09084 | 2.565 | 1.391 | 1.373 | 1.766 | 0.535 | 1.270 | 1.038 | 1.309 |
| IHW09092 | 2.855 | 1.477 | 1.450 | 1.568 | 0.508 | 1.062 | 0.985 | 1.095 |
| IHW09096 | 3.079 | 1.566 | 1.579 | 1.518 | 0.513 | 0.970 | 0.994 | 0.999 |
| IHW09098 | 2.754 | 1.470 | 1.532 | 1.473 | 0.556 | 1.002 | 1.078 | 1.033 |
| IHW09099 | 2.551 | 1.334 | 1.231 | 1.216 | 0.483 | 0.912 | 0.936 | 0.940 |
| IHW09101 | 2.917 | 1.479 | 1.728 | 1.538 | 0.592 | 1.040 | 1.148 | 1.072 |
| IHW09104 | 3.033 | 1.442 | 1.566 | 1.421 | 0.516 | 0.985 | 1.001 | 1.015 |
| IHW09105 | 2.689 | 1.394 | 1.303 | 0.933 | 0.485 | 0.669 | 0.940 | 0.690 |
| IHW09106 | 2.940 | 1.451 | 1.467 | 1.389 | 0.499 | 0.958 | 0.967 | 0.987 |
