# Supplementary material for: Simple and Versatile Molecular Method of Copy-Number Measurement Using Cloned Competitors
Source: PLoS One. 2013 Jul 30;8(7):e69414. doi: 10.1371/journal.pone.0069414 (PMC3728337; doi:10.1371/journal.pone.0069414)
Supplement: Methods S1 — Detailed mrcPCR procedure. (DOCX) [file pone.0069414.s010.docx]

Methods S1. Detailed mrcPCR procedure

1. Cloning of competitor sequences

a. Target or reference sequence is amplified with specific primers (Tables S2-S4), and cloned into a cloning vector (pGEM-T easy, Promega). The primers are not contained SNP sites of the SNP database (<http://genome.ucsc.edu/>). The sequences are not contained Sal*I* restriction enzyme site.

b. Introduction of base change into cloned target or reference sequences with Site-Directed Mutagenesis kit (Stratagene). For the *FCGR3A/B* copy-number evaluation, two base changes were introduced into the *IGF1* competitor

c. Digestion of cloned competitor plasmids with restriction enzyme (Sal*I*, Fermentas) and purification of digested plasmids with AxyPrep PCR Clean-up kit (Axygen)

d. Mixing and dilution of competitors into 10-50 fg/μl with distilled water (Gibco)

2. Competitive PCR reaction

a. Extra-denaturation: Denaturation of mixture of genomic DNA (10 ng), competitors (10-50 fg), and primers (5 pmole) at 98°C for 5 min in 10 μl volume

b. Addition of the other PCR components including PCR buffer II (Roche), MgCl_2_ (Roche, final 2.5 mM), dNTP mixture (Roche, final 0.25 mM), and Taq polymerase (Intron Biotechnology, SungNam, Korea; final 1.5 Unit) in 10 μl volume

c. PCR amplification under following conditions: denaturation at 94°C for 5 min, followed by 32 cycles of 10 s at 95°C, 30 s at 58°C, and 30 s at 72°C

d. Removal of remnant primers with AxyPrep PCR Clean-up kit (Axygen)

e. Electrophoretic analysis on 5% NuSieve agarose gel (Lonza) to ensure PCR amplification

3. Single-base-extension reaction to discriminate amplified sequences from genomic and competitor sequences

a. Single-base extension with SNaPshot multiplex kit (Applied Biosystems), extension primers (Tables S2 and S3), and 1 μl of cleaned PCR products with 15 cycles of 10 s at 96°C, 5 s at 50°C, and 30 s at 60°C

b. Treatment of alkaline phosphatase (Roche) for 15 min at 37°C to remove un-incorporated fluorescently labeled nucleotides

c. Separation of single-base extended primers in automatic sequencer (ABI 3100, Applied Biosystems) with POP6 polymer (Applied Biosystems), and analysis of peak heights with GeneScan software (ver. 3.7, Applied Biosystems).
